# Differential effects of environmental enrichment and physical exercise on glial biology in aging and aging-related conditions: a systematic review

**DOI:** 10.3389/fncel.2026.1859424

**Published:** 2026-07-28

**Authors:** Gaurav Singhal, Bernhard T. Baune

**Affiliations:** 1 Department of Otolaryngology, University of Wisconsin–Madison, Madison, WI, United States; 2 Department of Mental Health, University of Münster, Münster, Germany

**Keywords:** astrocytes, brain aging, environmental enrichment, glial cells, microglia, neuroimmune interactions, neuroinflammation, physical exercise

## Abstract

**Background:**

Aging is associated with progressive changes in glial cell dynamics, including altered morphology, activation states, and neuroimmune interactions of microglia, astrocytes, and other glial populations. These changes contribute to chronic neuroinflammation, impaired brain homeostasis, and increased vulnerability to cognitive decline and neurodegenerative disorders. Non-pharmacological lifestyle interventions such as environmental enrichment (EE) and physical exercise (PE) have shown promise in modulating brain aging, but their comparative and combined effects on glial cells remain incompletely understood.

**Objectives:**

This systematic review aimed to synthesize and compare the effects of EE, PE, and their combination on glial cell dynamics during aging. Specific aims included evaluating their individual and combined impacts on microglial and astrocytic morphology and function, identifying molecular mechanisms and neuroimmune crosstalk, benchmarking experimental paradigms, and examining regional, temporal, and lifespan variations in outcomes.

**Methods:**

A systematic search was conducted in PubMed, Scopus, and Google Scholar up to November 2025, following PRISMA 2020 guidelines. Preclinical (primarily rodent) studies were included if they examined well defined EE (cognitive, sensory, and social stimulation), isolated PE, or combined interventions in physiological aging models or in disease, injury, or stress paradigms considered relevant to aging because they shared glial mechanisms such as chronic neuroinflammation or impaired cellular homeostasis. These model classes were interpreted separately during synthesis, and studies were required to report glial relevant outcomes. A structured risk-of-bias assessment using the SYRCLE tool was conducted. Data were narratively synthesized due to anticipated heterogeneity.

**Results:**

Included studies showed that EE is consistently associated with increase in microglial number and morphological complexity and modulates peripheral T cell subsets, with stronger effects observed after long-term exposure. In contrast, PE more consistently reverses age-related microglial gene expression changes and induces region-specific remodeling of astrocytic morphology. Combined EE+PE interventions produced additive benefits on neurogenesis but yielded variable and non-superior effects on glial modulation. Molecular pathways such as BDNF-TrkB signaling and inflammatory cascades mediated these effects, with neuroimmune crosstalk (particularly involving peripheral T cells) influencing central glial states. Methodological heterogeneity and limited sex-specific analyses constrained generalizability.

**Conclusion:**

Environmental enrichment and PE exert distinct yet partially overlapping effects on glial plasticity and neuroinflammation across physiological aging and aging relevant pathological contexts, with EE showing greater strength in modulating glial-immune interfaces and PE in metabolic/anti-inflammatory glial remodeling. Combined interventions do not consistently outperform single modalities for glial outcomes.

## 1 Introduction

Research examining the comparative effects of environmental enrichment (EE), physical exercise (PE), and their combination on brain function and glial cell dynamics during aging has gained increasing importance considering the growing prevalence of age-related cognitive decline and neurodegenerative disorders. Aging is accompanied by progressive dysregulation of neural plasticity, immune homeostasis, and metabolic support (Alzheimers Dementia, 2025; Valles et al., 2019; Garcia-Dominguez, 2025), processes in which glial cells, particularly microglia and astrocytes, play a central role as mediators of neuroimmune interactions, synaptic remodeling, and brain resilience (Kwon and Koh, 2020; Matejuk and Ransohoff, 2020; Hanslik et al., 2021; Liu et al., 2023).

Microglia and astrocytes undergo significant age-related changes, including morphological alterations, heightened proinflammatory reactivity, senescence-like phenotypes, and impaired supportive functions, which contribute to chronic neuroinflammation, reduced neurogenesis, and cognitive impairment (Rozovsky et al., 1998; Nagele et al., 2004; Mrak and Griffin, 2005; Mansour et al., 2008; Bernal and Peterson, 2011; Tremblay et al., 2012; Kohman et al., 2013b; von Bernhardi et al., 2015; Clarke et al., 2018; Singhal et al., 2020b; Verkhratsky, 2025). These glial shifts are increasingly recognized as central drivers of age-related brain vulnerability and neurodegeneration. Aging also profoundly affects the oligodendrocyte lineage, including mature oligodendrocytes and their precursors (OPCs), leading to myelin degradation, reduced remyelination capacity, white matter atrophy, and impaired axonal support, resulting in slowed neural conduction, cognitive slowing, and increased susceptibility to neurodegenerative processes (Sloane et al., 2003; Peters, 2009; Miyamoto et al., 2013; Sams, 2021; Zhang et al., 2021; Kaya et al., 2022; Dimovasili et al., 2023; Groh and Simons, 2025). While less studied than microglia and astrocytes in the context of lifestyle interventions, emerging evidence indicates that both EE and PE can modulate oligodendrocytes dynamics (Tomlinson et al., 2016), with PE showing more consistent effects in delaying age-related white matter demyelination and myelin loss (Chen et al., 2020; Graciani et al., 2023; Butt et al., 2024), and EE promoting OPC proliferation, myelin remodeling, or preservation in stimulated contexts (Goldstein et al., 2021; Gao et al., 2022; Nicholson et al., 2022), potentially through overlapping yet distinct mechanisms (e.g., activity-dependent vs. experience-driven plasticity) (Forbes and Gallo, 2017; Goldstein et al., 2021; Nicholson et al., 2022; Butt et al., 2024).

Lifestyle interventions such as EE (providing cognitive, sensory, and social stimulation) and PE (structured or voluntary motor activity) have emerged as potent non-pharmacological modulators of brain aging. In early studies, both EE and PE enhanced hippocampal neurogenesis, synaptic plasticity, and cognitive performance in animal models (Singhal et al., 2014, 2019a,2019b; Zarif et al., 2018). More recent work has shifted focus to their effects on glial biology, revealing that microglia and astrocytes serve as key intermediaries in these benefits, responding to environmental and activity dependent cues by shifting toward homeostatic or neuroprotective states (Ehninger and Kempermann, 2003; Singhal et al., 2020a,^2021^; Guo et al., 2022; Verkhratsky, 2025). Despite overlapping mechanisms (e.g., neurotrophic signaling, reduced inflammatory tone), EE and PE appear to engage partially distinct pathways in glial modulation. EE often influences experience-dependent glial plasticity and neuroimmune crosstalk, while PE preferentially drives activity-dependent metabolic and anti-inflammatory effects on glia (Kelly, 2018; Ali et al., 2019; Mee-Inta et al., 2019; Singhal et al., 2020a,^2021^; Maugeri et al., 2021; Gao et al., 2022; Strohm and Majewska, 2024).

However, many EE paradigms incorporate running wheels or locomotor elements, confounding attribution of effects and limiting direct comparisons. Evidence on combined EE+PE interventions remains inconsistent, with some studies suggesting additive benefits on glial outcomes (Prado Lima et al., 2018) and others indicating context-dependent differences (Robison et al., 2020; Singhal et al., 2020a,^2021^). A comprehensive comparative analysis focused specifically on glial aging, addressing differential effects on microglial morphology and activation, astrocytic remodeling, oligodendrocytes/myelin dynamics, regional specificity, age-at-intervention timing, duration dependence, and potential neuroimmune peripheral interactions is currently lacking. This gap hinders the translation of findings into optimized, evidence-based lifestyle strategies for promoting glial health and cognitive longevity in aging.

The present systematic review aims to synthesize preclinical (and where available, clinical) evidence comparing EE, PE, and their combination on glial cell dynamics during aging. By prioritizing studies with clear glial endpoints (e.g., morphology, marker expression, inflammatory profiles, myelin integrity), we seek to clarify independent and interactive mechanisms, highlight methodological considerations (e.g., separation of cognitive-sensory vs. physical components), and identify priorities for future research. A detailed mechanistic overview of glial aging is provided to contextualize the interpretation of EE and PE effects; however, the primary focus of this review is the comparative synthesis of intervention-driven changes in glial outcomes.

The comparative interpretation of EE and PE effects in this review depends on understanding how each glial population changes with age. Aged microglia progressively develop a dystrophic phenotype, with retracted and fragmented processes, accumulated phagocytic inclusions, and lipofuscin deposition, alongside a primed state of elevated baseline pro-inflammatory cytokine expression and exaggerated responses to peripheral immune challenge (Conde and Streit, 2006; Sparkman and Johnson, 2008; Norden and Godbout, 2013; Ashraf et al., 2019). Astrocytes undergo soma enlargement, process thickening, and a transcriptional shift toward complement and inflammation associated reactive states that is most pronounced in hippocampus and striatum, with reduced expression of neurotrophic factors (BDNF, VEGF, FGF-2) and impaired glycogen and mitochondrial metabolism (Bernal and Peterson, 2011; Clarke et al., 2018; Hanslik et al., 2021; Sikora et al., 2021). Oligodendrocyte lineage cells lose maturation capacity, accumulate senescence markers (CDKN1A, CDKN2A) and integrated stress response signaling, and support shorter, thinner remyelinating internodes, with periventricular and frontal white matter particularly vulnerable (Sloane et al., 2003; Peters, 2009; Sams, 2021; Schlett et al., 2023; Windener et al., 2024). These cell type specific trajectories are amplified by glial senescence (SASP, SA-β-gal, lipid droplet accumulation) and by age related disruption of homeostatic axes such as CX3CL1-CX3CR1 and TGF-β Smad3, establishing the inflammatory tone against which EE and PE act (von Bernhardi et al., 2015; Sikora et al., 2021; Liu et al., 2025). This review evaluates how EE and PE interact with each of these processes.

While the primary focus of this review is on aging-related changes in glial biology, the available literature includes a substantial proportion of studies conducted in disease, injury, or stress-related models that share mechanistic features with aging, such as chronic neuroinflammation, glial activation, and impaired cellular homeostasis. In this review, these paradigms were treated as aging relevant but not biologically equivalent to physiological aging. Accordingly, findings were interpreted with explicit attention to model context, distinguishing normative aging studies from disease, injury, and stress paradigms wherever possible.

## 2 Methods

This systematic review was conducted and reported in accordance with the PRISMA 2020 guidelines (Page et al., 2021). Since this review was designed as a preclinical, mechanism focused synthesis spanning heterogeneous interventions, model systems, and glial endpoints, prospective registration was not completed before screening began. To improve transparency and reduce bias, the search strategy, eligibility criteria, screening process, extracted variables, and risk-of-bias methods are reported explicitly below.

The applicable PRISMA 2020 checklist items were followed (see Supplementary Table 1 for the completed checklist). Since no quantitative meta-analysis was performed due to anticipated heterogeneity, narrative synthesis was planned.

### 2.1 Search and selection process

A comprehensive search of PubMed, Scopus, and Google Scholar was performed. The search was last updated in November 2025. No language or publication date restrictions were applied during the initial electronic searches, but only English language peer reviewed articles were considered at the full text eligibility stage.

Titles and abstracts were screened for relevance, followed by full text review of potentially eligible articles. Screening was primarily conducted by one reviewer (GS). A subset of records was independently reviewed by a second reviewer (BB) to ensure consistency. Any discrepancies were resolved through discussion. Additional articles were identified by reviewing reference lists of included studies. The full electronic search strategy for PubMed (including exact terms and Boolean operators) is provided in Supplementary Table 2. For Google Scholar searches, results were screened in order of relevance, and the first 200 records were evaluated for eligibility. Duplicate records identified across databases were removed prior to full-text screening.

**FIGURE 1:**
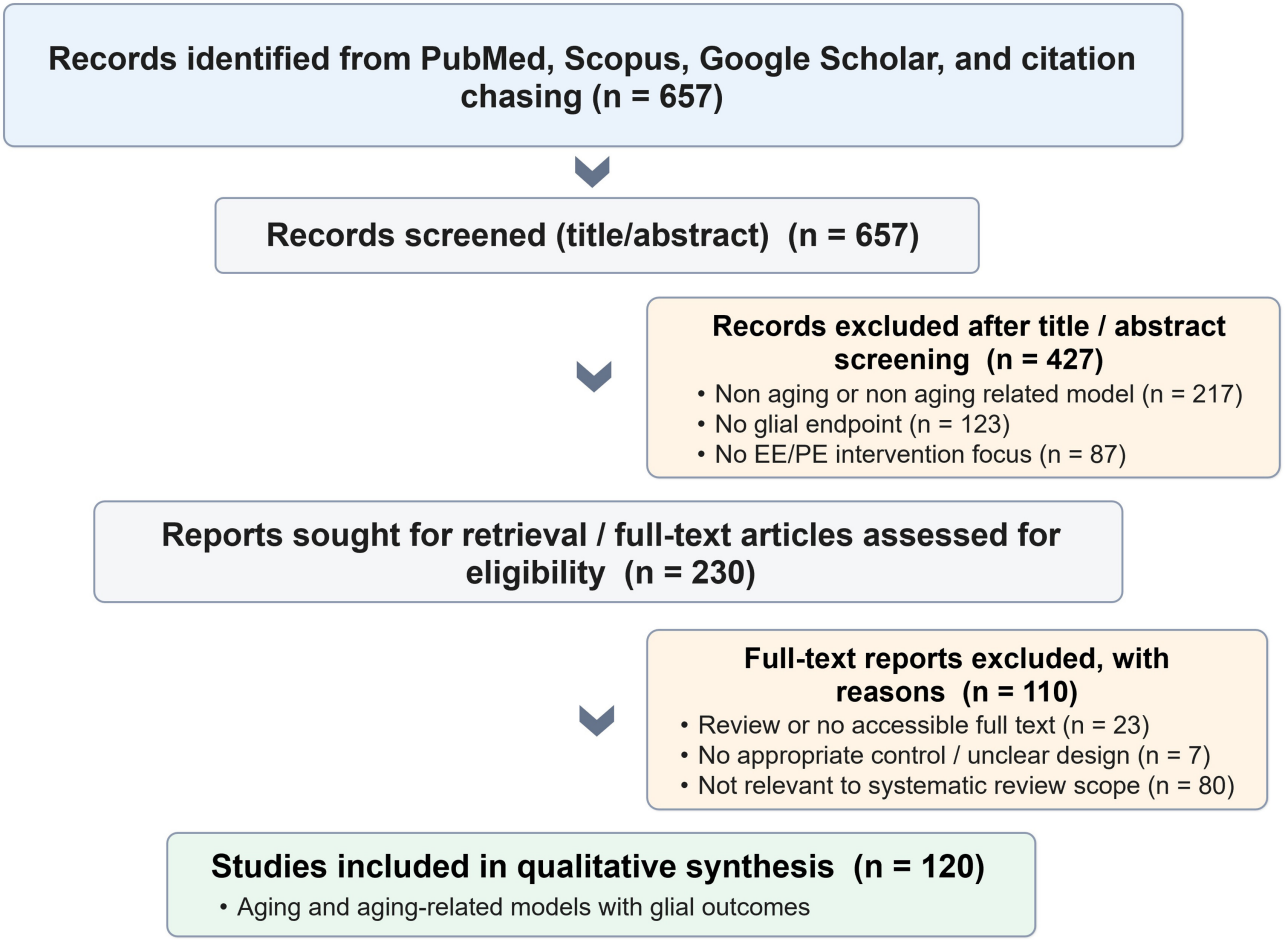
Study inclusion flowchart. Methodology for search and collection of relevant articles for this review, following PRISMA guidelines.

This process yielded a total of 657 records for initial review. After applying the inclusion and exclusion criteria, 120 articles were included in the review (Figure 1).

### 2.2 Data extraction

Data were extracted independently by GS who performed initial extraction of key items, including study design, population (species, strain, age at intervention), intervention details (EE/PE type, duration, components), comparator, primary glial outcomes (e.g., markers, brain regions, quantitative/qualitative results), secondary outcomes, and main findings. A second reviewer (BB) verified all extracted data for accuracy and completeness. No attempts were made to contact study authors for additional data, as all necessary information was available in published reports. When included studies used legacy binary terms such as M1/M2 microglia or A1/A2 astrocytes, these labels were interpreted cautiously and are described in this review, where possible, using marker, morphology, or function-based terminology rather than assuming fixed polarized states.

### 2.3 Eligibility criteria

Eligibility criteria were defined using a modified PICOS framework to ensure transparency and reproducibility:

Population: Aging or aged animal models (primarily rodents, including mice and rats). Limited inclusion of human studies only if they directly reported glial cell endpoints in the context of aging and lifestyle interventions.
Intervention: Environmental enrichment (EE), defined as paradigms providing cognitive, sensory, and social stimulation (CSS), either with or without a physical exercise component (e.g., running wheels); and/or physical exercise (PE), defined as voluntary or forced aerobic/motor activity (e.g., wheel running, treadmill exercise).
Comparator: Standard housing conditions or sedentary control groups without enrichment or structured exercise.
Primary outcomes: Glial cell markers and dynamics, including microglial number, morphology, activation state, and marker expression (e.g., Iba1, CD68, CD11b), astrocytic number, morphology, reactivity, and marker expression (e.g., GFAP), and oligodendrocyte lineage or myelin-related measures (e.g., OPC proliferation, myelin integrity). Since many included studies predated widespread use of more microglia restricted markers such as Tmem119 or P2ry12, microglial outcomes were accepted when based on commonly used markers in conjunction with morphology, anatomical localization, and study context. A small number of more recent aging studies have begun incorporating Tmem119 or single cell annotation level under EE or PE (Ali et al., 2019; Chauquet et al., 2024; Perez et al., 2024), but chronic protein level Tmem119 or P2ry12 quantification has not been reported in the aged EE/PE literature yet, and we did not identify aged EE/PE studies directly reporting P2ry12 endpoints. CD68 was interpreted here as a lysosomal/phagocytic myeloid marker rather than a microglia specific identifier, and Iba1 only outcomes were interpreted more cautiously as reflecting microglial or broad brain myeloid labeling unless additional marker or anatomical information supported microglial attribution.
Secondary outcomes: Neuroimmune interactions (e.g., peripheral immune cell profiles influencing central glia), neuroplasticity endpoints (e.g., neurogenesis, synaptogenesis, synaptic protein expression, neurotrophic factor signaling such as BDNF or NGF), and behavioral measures directly linked to glial function (e.g., learning, memory, cognition, anxiety-like, or depressive-like behavior).
Study design: Experimental (primarily preclinical), peer-reviewed original research articles published in English.

Priority was given to studies employing well defined EE paradigms and isolated PE interventions, as well as those addressing age dependent or lifespan effects of EE and/or PE on glial biology. Supplementary citation searching of reference lists from included studies and key reviews was performed to identify additional relevant articles. In addition to physiological aging models, studies involving aging-relevant disease, injury, or stress paradigms were included only when they exhibited glial alterations overlapping with aging-related mechanisms (e.g., neuroinflammation, senescence-like phenotypes, or impaired glial homeostasis). These studies were retained to contextualize aging relevant pathways but were not considered interchangeable with normative aging models.

Studies were excluded if they focused primarily on non-neural outcomes (e.g., metabolic, cardiovascular, or peripheral musculoskeletal adaptations) without assessment of brain or glial endpoints, used poorly defined, minimally implemented, or confounded EE or PE paradigms (e.g., uncontrolled environmental variables), lacked appropriate control groups, randomization, or clear experimental design, and were opinion pieces, conference abstracts, case reports or editorials. Of the records screened at full text stage, 23 were excluded because the full text could not be retrieved despite institutional subscription access at both author institutions and inter library loan requests. These records were checked for open access versions, PubMed Central deposits, and author uploaded manuscripts before exclusion.

### 2.4 Data synthesis

Given the anticipated methodological heterogeneity across studies (e.g., differences in animal models, intervention protocols, glial markers, brain regions, and outcome measures), no quantitative meta-analysis was planned or performed. Instead, a narrative synthesis was conducted to summarize and compare the effects of EE, PE, and combined interventions on glial aging outcomes. Findings are organized by glial cell type (microglia, astrocytes, oligodendrocytes where reported), intervention type, and model context (physiological aging vs. disease/neurodegenerative vs. injury/stress paradigms). Where possible, subgroup considerations include intervention timing (short- vs. long-term), brain region specificity, age dependent effects, and whether outcomes arose from normative aging or aging relevant pathological contexts. Tables in the manuscript summarize study characteristics, key findings, and risk of bias. The synthesis highlights consistencies, inconsistencies, and evidence gaps without formal statistical pooling.

### 2.5 Risk of bias assessment

The methodological quality and risk of bias of all included preclinical animal studies were independently assessed using the SYRCLE Risk of Bias tool for animal studies (Hooijmans et al., 2014), a validated instrument specifically adapted from the Cochrane Risk of Bias tool for application to *in vivo* animal experiments. This tool evaluates ten bias domains (D) across six categories (Supplementary Table 3).

Each domain was assessed and assigned one of three ratings: Low Risk of Bias (L), indicating adequate methodological safeguards were described and employed; High Risk of Bias (H), indicating an identifiable methodological flaw or quality concern; or Unclear Risk of Bias (U), indicating insufficient reporting to permit a definitive judgment. An Unclear rating was not interpreted as equivalent to confirmed bias, but rather as reflecting a structural limitation of preclinical reporting norms prevalent in the neuroscience literature, particularly for studies published prior to the widespread adoption of animal reporting standards such as ARRIVE 2.0. The SYRCLE assessment was applied to all study entries across four evidence categories: (i) EE effects on microglia (*n* = 8 studies; Table 1), (ii) EE effects on astrocytes (*n* = 11 studies; Table 2), (iii) PE effects on microglia (*n* = 12 studies; Table 3), and (iv) PE effects on astrocytes (*n* = 12 studies; Table 4), totaling 43 assessed study entries. Two studies appeared in both Tables 1, 3, Williamson et al. (2012), Singhal et al. (2020a) and were assessed independently for each outcome category. Outcome specific tabulated SYRCLE summaries were retained rather than a single pooled visual summary because several studies contributed to more than one outcome category, and outcome level reporting provided the clearest representation of bias judgments across the included evidence base.

**TABLE 1:** Summary of findings for environmental enrichment effects on microglial aging.

Study	Model/age	Study context	Exposure	Physical component included in EE	Region	Structural change	Activation/ functional state	Aging/ senescence relevance	Main finding
Ali et al., 2019	Mouse, C57BL/6 (M/F); middle age, 10 mo	Normal aging	Long-term EE, 7.5–12 mo	Yes	HC, Hy, A	Hypertrophy with increased ramification; no increase in density	Reduced neuroinflammatory cytokines and MHC-II	Attenuated aging-associated dysfunction; no dystrophic accumulation	Middle-age EE induced a non-aged microglial phenotype across multiple regions.
de Oliveira et al., 2020	Mouse, Swiss albino (F); weaning to 20 mo	Normal aging	Lifelong EE	Yes	DG molecular layer	Reduced morphologic diversity; convergence toward an intermediate complex phenotype	Suggests stabilization toward homeostatic surveillance	Adaptive rather than degenerative microglial aging	Lifelong EE constrained age-related diversification of microglial morphotypes.
Xu et al., 2016	Mouse, WT C57BL/6 (M/F); young adult	Disease model (Aβ)	Prolonged EE before/after Aβ oligomers	Yes	DG	Increased branching complexity; normalized inflammatory morphology	Prevented Aβ-induced inflammatory gene expression and cytokine release	Prevented inflammaging-like priming in an AD-relevant context	EE suppressed inflammatory responses to Aβ and preserved immune homeostasis.
Williamson et al., 2012	Rat, Sprague-Dawley (M); adult	Normal aging (immune challenge component)	EE, 7 wk, 12 h/day; ±LPS	Yes	DG	Increased Iba1 antigen expression in DG	Blunted cytokine/chemokine response to LPS	Reduced inflammatory vulnerability	EE altered immune responsiveness and improved resistance to inflammatory challenge.
Singhal et al., 2021	Mouse, C57BL/6 (M/F); middle age, 8–13 mo	Normal aging	Short-term EE, 7 wk	No	DG, CA1, CA3	Increased Iba1+ microglia in DG (strongest in EE)	Not inflammatory; no astroglial inflammatory signature	Modulates age-related glial dynamics without pathology	Short EE changed microglial abundance more strongly than PE or EE + PE.
Singhal et al., 2020a	Mouse, C57BL/6; middle age	Normal aging	Short vs. long-term EE	No	DG, CA1, CA3	Increased microglial number regardless of duration	No evidence of pro-inflammatory activation	Not directly assessed	EE increased microglial number, whereas other glial/immune outcomes depended more on duration.
Stuart et al., 2019	Mouse, APP/PS1 (M); late life, 12–18 mo	Disease model (AD)	Late-life EE, 6 mo	Yes	HC, neocortex	Reduced microglial burden; increased CD68+ phagocytic cells	Shifted toward a functional phagocytic phenotype	Attenuated AD-associated dysregulation	Late-life EE preserved memory and reduced pathologic microglial accumulation without changing Aβ burden.
Li et al., 2025	Mouse, C57BL/6J (M); aged, 18 mo	Stress/injury (social isolation + POCD)	EE for 4 wk under social isolation	Yes	Hippocampus, especially CA1	Morphology not primary endpoint; lower Iba1	Reduced pro-inflammatory markers iNOS and CD86, and lowered inflammatory cytokines	Not directly assessed	In socially isolated aged mice, EE improved cognition and reduced hippocampal neuroinflammation.

mo, months; wk, weeks; EE, environmental enrichment; PE, physical exercise; EE+PE, combined environmental enrichment and physical exercise; HC, hippocampus; Hy, hypothalamus; A, amygdala; DG, dentate gyrus; CA1/CA3, cornu ammonis subfields 1 and 3; Iba1, ionized calcium-binding adaptor molecule 1; GFAP, glial fibrillary acidic protein; MHC-II, major histocompatibility complex class II; Aβ, amyloid beta; LPS, lipopolysaccharide; IL, interleukin; TNF, tumor necrosis factor; CD68, CD86, cluster of differentiation markers; iNOS, inducible nitric oxide synthase; WT, wild type; APP/PS1, amyloid precursor protein/presenilin-1; POCD, postoperative cognitive dysfunction; M/F, males and females. Study context classifications include normal aging, disease models (e.g., AD), and stress/injury-related paradigms. The “Physical component included in EE” column indicates whether enrichment paradigms contained locomotor elements (e.g., running wheels) or were explicitly wheel-free.

**TABLE 2:** Summary of findings for physical exercise effects on microglial aging.

Study	Model/age	Study context	Exposure	Region	Structural change	Activation/functional state	Aging/senescence relevance	Main finding
Barrientos et al., 2011	Rat, F344 x BN (M); aged 22–24 mo	Normal aging	Voluntary wheel running, 6 wk	HC, CA1	Not morphology-focused	Reduced IL-1β and IL-6; increased IL-1RA	Not reported directly	Running counteracted age-related hippocampal inflammation and rescued memory-related outcomes.
Mela et al., 2020	Mouse, C57BL/6; aged 18 mo	Normal aging	Treadmill, 10 d	HC and cortex	Noted restoration toward resting morphology after culture; increased phagocytic capacity	Reduced inflammatory/metabolic dysregulation	Lowered beta-galactosidase and p16INK4A	Brief treadmill training reduced senescence markers and reprogrammed aged microglia.
Rodriguez et al., 2015	Mouse, 3xTg-AD (M); chronic from 3 to 12 mo	Disease model (AD)	Voluntary running vs. ENR vs. RUN+ENR	HC CA1	RUN caused hypertrophy; ENR reduced numerical density	Combined exposure prevented AD-associated density increase	Not reported	Exercise and enrichment shaped microglia differently: running promoted hypertrophy, enrichment reduced burden.
Yoo et al., 2015	Rat, ZDF/ZLC (M); intervention from 30 wk	Disease model (metabolic/diabetes)	Treadmill, 10 wk	HC	Reactive morphology in sedentary diabetic rats shifted toward ramified resting-like morphology after exercise	Reduced TNF-alpha, IL-6, and IL-1β	Not assessed	Exercise reduced reactive microgliosis and inflammatory cytokines in diabetic aging-related pathology.
Vukovic et al., 2012	Mouse, C57BL/6 background (F); aged 9–20 mo comparison	Normal aging	Voluntary running, 2–4 wk	HC, DG/SGZ	Not focused on gross morphology	Reduced MHC II+ microglia; increased CX3CL1-CX3CR1 signaling	Aging reduced CX3CL1 and NPC activity; running restored them	Exercise promoted a neuroprotective microglial state linked to neurogenesis.
Kohman et al., 2012	Mouse, C57BL/6; aged 18 mo	Normal aging	Voluntary wheel running, ∼6 wk	HC (DG)	Reduced microglial proliferation; no detailed morphology	Shift toward proneurogenic microglial phenotype	Attenuation of age-related microglial priming	Exercise reduced microglial proliferation and promoted a supportive phenotype linked to neurogenesis.
Kohman et al., 2013b	Mouse, BALB/c (M/F); aged 21–22 mo	Normal aging	Voluntary wheel running, 10 wk	HC and whole brain minus HC	Not morphology-focused	Reduced MHC II and CD86 especially in aged females	Not assessed	Exercise attenuated basal pro-inflammatory activation in a sex- and region-dependent manner.
Krejcova et al., 2024	Rat, Wistar (M); aged ∼23 mo	Normal aging (early-life adversity modifier)	Late-life treadmill, 5 wk	DG layers	Reduced age-related microglial accumulation	Shift toward less activated/more homeostatic profile inferred from density changes	Not assessed	Late-life treadmill attenuated microgliosis in aged males despite early-life adversity.
Lu et al., 2017	Rat, Sprague-Dawley (M); STZ-induced AD model	Disease model (AD-like; STZ-induced)	Treadmill, 4 wk	HC, mainly CA1	No detailed morphometrics	Suppressed pro-inflammatory markers/cytokines and promoted reparative associated microglial signaling.	Not assessed	Exercise shifted microglia from pro-inflammatory toward reparative-associated states in an AD-like model.
Chauquet et al., 2024	Mouse, C57BL/6 (F); aged 18 mo	Normal aging	Voluntary running, 21 d plus 14 d rest	HC	Transcriptomic rather than morphologic endpoint	Reversed about 90% of aging-associated microglial DEGs; reduced DAM-like states	Functional rejuvenation rather than classical senescence assay	Exercise restored a young-like homeostatic transcriptomic profile in aged microglia.
Littlefield et al., 2015	Mouse, C57BL/6J (M); aged 22 mo	Normal aging (immune challenge component)	Voluntary running, 9 wk ± LPS	DG	Not morphology-focused	Increased proportion of BDNF+ microglia and buffered LPS response	Addresses age-related priming rather than direct senescence markers	Exercise promoted a neuroprotective phenotype that limited inflammation-driven decline.
Liu et al., 2024	Mouse, C57BL/6J (M); D-gal aging model	Accelerated aging (D-gal model)	Aerobic treadmill, 8 wk	HC	Reduced Iba1+ density and optical density	Suppressed NF-kB and MAP3K5/JNK inflammatory signaling	Model of accelerated inflamm-aging	Exercise reduced reactive microgliosis and inflammatory cascades while preserving neuronal viability.

mo, months; wk, weeks; d, days; PE, physical exercise; RUN, voluntary running; HC, hippocampus; DG, dentate gyrus; CA1, cornu ammonis subfield 1; Iba1, ionized calcium-binding adaptor molecule 1; GFAP, glial fibrillary acidic protein; IL, interleukin; TNF, tumor necrosis factor; BDNF, brain-derived neurotrophic factor; MHC II, major histocompatibility complex class II; CD86, CD206, cluster of differentiation markers; ARG1, arginase-1; NF-κB, nuclear factor kappa B; MAP3K5, mitogen-activated protein kinase 5; JNK, c-Jun N-terminal kinase; M/F, males and females. Study context classifications include normal aging, accelerated aging (e.g., D-galactose models), disease models (e.g., AD, metabolic disorders), and immune challenge paradigms (e.g., LPS exposure), distinguishing physiological aging from pathology-driven glial responses.

**TABLE 3:** Summary of findings for environmental enrichment effects on astroglial aging.

Study	Model/age	Study context	Exposure	Physical component included in EE	Region	Structural change	Activation/ functional state	Aging/ senescence relevance	Main finding
Sampedro-Piquero et al., 2014	Rat, Wistar (M); aged, 18 mo	Normal aging	Intermittent EE, 2 mo, 3 h/day	Yes	Dorsal HC: CA1, CA3, DG	Increased GFAP+ cells in DG/CA3 and greater arbor complexity	Supports astrocytic plasticity/brain reserve	Relevant to aged hippocampal adaptation	EE increased astrocyte complexity in parallel with better spatial memory.
Buchhold et al., 2007	Rat, Sprague-Dawley; young and aged post-stroke	Disease/injury (stroke)	Post-stroke EE, 4 wk	Yes	Cortex/peri-infarct border zone	Reduced glial scar volume and infarct volume	Suggests improved tissue-repair milieu	Relevant to age-related post-injury astroglial response	EE improved functional recovery and reduced astrocytic scar burden.
Beauquis et al., 2013	Mouse, PDAPP-J20 females; pathology from 5 to 8 mo	Disease model (AD)	EE, 3 mo	No	HC CA1	Prevented plaque-associated hypertrophy and normalized non-plaque astrocyte volume	Lowered soluble Aβ1-40 and Aβ1-42	Protective against early pathology-linked remodeling	Wheel-free EE preserved a more control-like astroglial phenotype in AD-prone mice.
Bento-Torres et al., 2017	Mouse, albino Swiss (F); prion model with aging comparison	Disease model (prion)	EE for 5 wk before inoculation	Yes	HC: DG, CA3	Altered astroglial morphologic profile but did not fully reverse disease-related change	Supportive functions not directly tested	Aging and disease altered astrocyte families	EE shifted astroglial morphology and delayed disease progression signs.
Singhal et al., 2021	Mouse, C57BL/6 (M/F); ∼5, 10, 15 mo endpoints	Normal aging	Short-term EE, 7 wk	No	DG	No change in astrocyte number	Supportive functions not assessed	Implies short EE may be insufficient for astrocyte changes	EE changed microglia but not DG astrocyte counts.
Cunha Feio, Leal et al., 2023	Mouse, Swiss albino (F); lifelong housing to 6 or 18 mo	Normal aging	Lifelong EE	Yes	HC CA1, CA3, DG	Astrocyte numbers varied with age and housing context; impoverished aging reduced CA1 radiatum density	Supportive functions not assessed	Highlights interaction of aging and environment	Astrocyte distribution changed with both age and environmental condition.
Soffie et al., 1999	Rat, male; lifelong EE to 23 mo	Normal aging	Lifelong EE	Yes	DG and CA1	Prevented age-related increase in GFAP+ astrocytes	Consistent with reduced astroglial reactivity burden	Directly relevant to aging-associated astroglial activation	Lifelong EE blunted the typical age-related rise in hippocampal astrocytes.
Diniz et al., 2010	Mouse, albino Swiss (F); 6 and 20 mo	Normal aging	Lifelong EE	Yes	DG layers	EE and aging both altered layer-specific GFAP+ distribution, especially molecular layer	Supportive functions not directly assayed	Shows layer-specific astrocyte plasticity with age	EE reshaped DG astrocyte distribution rather than simply reducing it.
Diniz et al., 2016	Mouse, albino Swiss (F); young adult and aged	Normal aging	Lifelong EE	Yes	DG molecular layer	Preserved complex astrocyte phenotypes and prevented simplification	Linked to synaptic coverage/metabolic support and better memory	Prevents age-related collapse of astrocyte diversity	Lifelong EE maintained complex astroglial phenotypes associated with cognitive resilience.
Williamson et al., 2012	Rat, Sprague-Dawley (M); adult	Normal aging (immune challenge component)	Daily EE, 7 wk, 12 h/day	Yes	DG primarily	Increased GFAP signal in DG, not CA1/CA3/cortex	Reduced cytokine response after LPS and altered BDNF mRNA	Relevant to region-specific glial modulation	EE selectively modified DG astroglial and neuroimmune responses.
Singhal et al., 2020a	Mouse, C57BL/6 (M/F); multiple age points	Normal aging	Short vs. long-term EE	No	DG; HC subfields in design	Long-term, not short-term, EE increased GFAP+ astrocytes in DG	Not direct functional assays	Indicates duration-sensitive astrocyte response	EE without wheels can modulate astrocytes, but sufficient duration is required.

mo, months; wk, weeks; EE, environmental enrichment; PE, physical exercise; HC, hippocampus; DG, dentate gyrus; CA1/CA3, cornu ammonis subfields 1 and 3; ML, molecular layer; GFAP, glial fibrillary acidic protein; Iba1, ionized calcium-binding adaptor molecule 1; Aβ, amyloid beta; LPS, lipopolysaccharide; BDNF, brain-derived neurotrophic factor; AD, Alzheimer’s disease; M/F, males and females. Study context classifications include normal aging, disease models (e.g., AD, prion disease), and injury-related paradigms (e.g., stroke). The “Physical component included in EE” column distinguishes wheel-free enrichment from enrichment paradigms that included locomotor activity.

**TABLE 4:** Summary of findings for physical exercise effects on astroglial aging.

Study	Model/age	Study context	Exposure	Region	Structural change	Activation/functional state	Aging/senescence relevance	Main finding
Belaya et al., 2020	Mouse, 5xFAD and WT littermates (M); exercise from 6 wk to 7 mo	Disease model (AD)	Long-term voluntary running, ∼6 mo	HC, subiculum	Increased GFAP+ astrocytes, soma hypertrophy, and greater branching selectively in plaque-associated astrocytes	Restored astrocytic BDNF expression and PSD-95 without altering Aβ burden or microglial activation	AD-related astroglial remodeling rather than normal aging alone	Exercise induced hypertrophic, BDNF-supportive astrocytes.
Cai et al., 2025	Mouse, 5xFAD and WT (M); exercise started at 5 mo	Disease model (AD)	Aerobic treadmill, 4 wk	HC	Reversed astrocytic mitochondrial structural damage and senescence-like phenotype	Improved mitochondrial membrane potential, reduced ROS, increased CD38, and enhanced astrocyte-to-neuron mitochondrial transfer	AD-associated astrocyte metabolic dysfunction with senescence-like features	Exercise restored astrocytic mitochondrial function.
Cao et al., 2022	Rat, Wistar (M); adult	Disease model (vascular aging-related)	Electric wheel running, 2 mo	Corpus callosum	Reduced C3+/GFAP+ reactive astrocytes and increased S100A10+/GFAP+ astrocytes associated with protective reactivity; no increase in total astrocyte number	Enhanced primary ciliogenesis and ciliary length; suppressed ERK/JNK signaling	Relevant to chronic cerebrovascular injury and maladaptive astroglial aging-like reactivity	Exercise shifted astrocytes toward a more protective reactive profile.
Chen et al., 2021	Mouse, C57BL/6 (F)	Injury model (ischemia)	Forced treadmill running, 2 wk	Ischemic cortex	Increased astrocyte hypertrophy and activation with a shift toward a synapse-supportive reactive state	Enhanced synaptogenic support through STAT3-Gpc6 signaling with increased PSD95 and reduced infarct volume	Relevant to injury-induced astroglial remodeling rather than baseline aging	Exercise promoted astrocyte-mediated synaptic repair.
He et al., 2017	Mouse, C57BL/6J and Thy1-GFP (M); aged 14-16 mo	Normal aging	Voluntary wheel running, 6 wk	Cortex; HC CA1, CA3, DG	Reduced age-related reactive astrogliosis with fewer GFAP+ astrocytes	Restored AQP4 expression and perivascular polarity, improving glymphatic CSF-ISF exchange and Aβ clearance	Directly relevant to age-associated astroglial activation and impaired clearance	Exercise reduced astrogliosis and improved clearance.
Kelty et al., 2022	Rat, Wistar (F), aged	Post-surgical LPS neuroinflammation model	Progressive resistance training, 3 wk	DG	Attenuated LPS-induced reactive astrocyte remodeling reflected by lower GFAP mRNA and protein	Reduced astrocyte-associated TNF-a and IL-1b expression with improved recognition memory	Relevant to inflammatory astroglial remodeling that may overlap with aging-related neuroinflammation	Exercise reduced astrocyte-driven inflammation.
Latimer et al., 2011	Mouse, C57BL/6 (F); middle-aged 11–13 mo	Normal aging	Voluntary wheel running, 6 wk	HC CA1 stratum radiatum	Reduced astrocyte hypertrophy/reactivity, including lower GFAP immunoreactivity and less stellate morphology	Associated with improved neurovascular environment and normalization of myelin-related markers	Directly relevant to midlife age-associated astrocyte reactivity	Exercise reversed astrocyte hypertrophy.
Li et al., 2024	Mouse, C57BL/6J (M)	Stress model (chronic unpredictable stress)	Forced treadmill running, 4 wk	HC CA1, CA2, CA3, DG	Restored astrocyte number and morphological complexity in CA2-3 and DG, with no significant change in CA1	Increased astrocytic GLT-1 expression and astrocyte-contacted PSD95+ synapses	Stress-induced astroglial simplification rather than direct senescence	Exercise restored astrocyte–synapse coupling.
Liu et al., 2023	Mouse, APP/PS1 and WT (M); 10 mo	Disease model (AD)	Voluntary wheel running, 3 mo	Medial PFC	Reduced astrocyte hyperplasia and S100B expression, but did not normalize hypertrophic morphology or C3 expression	Enhanced PSD95+ puncta contacting astrocyte protrusions, indicating improved astrocyte-synapse coupling	AD-related astrocyte pathology with persistent hypertrophy	Exercise reduced astrocyte hyperplasia.
Liu et al., 2024	Mouse, C57BL/6J and Thy1-YFP (M)	Accelerated aging (D-gal model)	Treadmill running, 18 d	Primary motor cortex	Normalized alcohol-induced astrocyte hypertrophy and reduced excessive process overgrowth without changing density	Restored physiological astrocytic calcium activity, improved astrocyte-neuron coupling, and supported motor learning	Relevant to maladaptive astrocyte hyperreactivity rather than baseline aging	Exercise normalized astrocyte function.
Tapia-Rojas et al., 2016	Mouse, APPswe/PS1dE9 and WT; 5 mo	Disease model (AD)	Voluntary wheel running, 10 wk	HC; cortex	Reduced astrogliosis, including lower GFAP immunoreactivity and smaller astrocyte soma size	Supportive functions not directly assayed; effects occurred alongside lower Aβ burden, reduced tau phosphorylation, and improved cognition	AD-related reactive astrogliosis	Exercise reduced reactive astrogliosis.
Yamaguchi et al., 2023	Mouse, C.B-17/Icr-+/+Jcl (M)	Injury model (stroke; MCAO)	Voluntary wheel running, 15 d after stroke	Peri-infarct motor cortex	Did not increase total GFAP+ astrocyte number, but increased persistence of newborn BrdU+/GFAP+ astrocytes	Reduced astrocytic Lcn2 expression and the proportion of Lcn2+/GFAP+ astrocytes, consistent with a less neurotoxic state	Relevant to post-injury astrocyte remodeling rather than baseline aging	Exercise increased newborn astrocytes and reduced toxicity.

mo, months; wk, weeks; d, days; PE, physical exercise; HC, hippocampus; DG, dentate gyrus; CA1/CA2/CA3, cornu ammonis subfields; mPFC, medial prefrontal cortex; M1, primary motor cortex; GFAP, glial fibrillary acidic protein; Iba1, ionized calcium-binding adaptor molecule 1; S100B, S100 calcium-binding protein B; S100A10, S100 calcium-binding protein A10; GLT-1, glutamate transporter-1; BDNF, brain-derived neurotrophic factor; TrkB, tropomyosin receptor kinase B; AQP4, aquaporin-4; MBP, myelin basic protein; VEGF, vascular endothelial growth factor; C3, complement component 3; ARG1, arginase-1; TGF-β, transforming growth factor beta; ERK, extracellular signal-regulated kinase; JNK, c-Jun N-terminal kinase; MAPK, mitogen-activated protein kinase; STAT3, signal transducer and activator of transcription 3; Gpc6, glypican-6; CD38, cluster of differentiation 38; ROS, reactive oxygen species; BrdU, 5-bromo-2′-deoxyuridine; Ki67, marker of proliferation protein Ki-67; Lcn2, lipocalin-2; Ca^2+^, calcium ion; AD, Alzheimer’s disease; MCAO, middle cerebral artery occlusion (experimental stroke model); D-gal, D-galactose-induced accelerated aging model; M/F, males and females. Study context classifications include normal aging, disease models, injury models (e.g., ischemia, MCAO), stress models, and accelerated aging paradigms, reflecting the predominance of non-physiological aging evidence in astrocyte-focused PE studies.

### 2.6 Definitions of interventions

To enable meaningful comparison across studies, interventions were categorized into two operational groups: EE, defined as paradigms providing cognitive, sensory, and social stimulation, with or without explicit locomotor components (e.g., running wheels); and PE, defined as paradigms involving voluntary or forced motor activity (e.g., wheel running or treadmill exercise) without additional cognitive-sensory enrichment elements. Given that many EE paradigms incorporate locomotor components, findings were interpreted cautiously, as the effects of cognitive-sensory stimulation could not always be clearly separated from those of physical activity.

## 3 Results

### 3.1 SYRCLE risk of bias assessment

Across the full set of included studies, D2 (Baseline characteristics) received a Low-Risk rating in 42 of 43 entries, reflecting the standard use of matched controls or littermates of consistent strain, age, and sex. One study (Buchhold et al., 2007) was rated Unclear due to the inclusion of both young and aged cohorts in a post-stroke model without explicit confirmation of baseline group equivalence. Domains D1, D3, D4, D5, D6, D7, and D9 received Unclear ratings uniformly across all entries, consistent with the established pattern of under-reporting in the preclinical literature rather than evidence of active bias. D8 (Incomplete outcome data) was rated Low Risk in 39 of 43 entries where complete group-level data were reported (four entries were rated Unclear where attrition was not explicitly addressed). D10 (Other bias) was rated Unclear in all entries, as no study demonstrated extreme sample size limitations, implausible outcomes, or other identifiable structural concerns.

The overall risk of bias profile across the included literature was considered moderate and broadly consistent with published preclinical systematic reviews in neuroimmunology and exercise neuroscience. The predominance of Unclear ratings across most SYRCLE domains reflects under reporting of methodological procedures rather than confirmed methodological deficiencies and should not be interpreted as evidence of high bias across the included literature.

### 3.2 Microglia

Microglial aging is dominated by progressive dystrophy (process retraction, fragmentation, phagocytic inclusions, lipofuscin) and a primed pro-inflammatory baseline that is driven by impaired TGF-β Smad3 anti-inflammatory signaling and dysregulated CX3CL1-CX3CR1 neuron microglia communication (Conde and Streit, 2006; Norden and Godbout, 2013; von Bernhardi et al., 2015; Ashraf et al., 2019; Liu et al., 2025). White matter microglia and region-specific subsets (e.g., primary visual cortex) show particularly pronounced morphological deterioration with age (Tremblay et al., 2012). Sex differences in microglial phagocytic capacity have also been reported, with aged female microglia losing context dependent phagocytic flexibility (Yanguas-Casas et al., 2020). EE related microglial outcomes are interpreted against this background.

#### 3.2.1 EE effects on microglia

The majority of studies examining the effects of EE on microglia in aging and aging-related models reported consistent changes in microglial morphology, density, and phenotypic states, with effects often more pronounced after long-term exposure and in middle-aged to aged rodents. To improve interpretability, findings from physiological aging models are distinguished below from those arising in disease, neurodegenerative, stress, or inflammatory challenge paradigms when such evidence is discussed.

• Morphology

Long-term EE (typically 6–12 months, started at middle age) was associated with increased microglial ramification and complexity in multiple brain regions, including the hippocampus, hypothalamus, and amygdala. For example, EE led to a shift toward more ramified (homeostatic-like) morphology compared to age-matched controls in standard housing, as assessed in the original studies by Iba1 immunohistochemistry and Sholl analysis or process length metrics (Ali et al., 2019). This contrasted with the typical aging-related de-ramification and reduced surveillance capacity observed in sedentary aged animals (Ali et al., 2019). Short-term EE (e.g., 4–8 weeks) produced more variable or region-specific morphological changes, with a study reporting modest increases in process complexity primarily in the dentate gyrus (de Oliveira et al., 2020).

In addition to increased ramification, a study has reported microglial hypertrophy and expansion of total process volume following prolonged EE, suggesting an enhancement of territorial coverage and environmental surveillance rather than a reactive transformation (Ali et al., 2019). Importantly, these EE-induced morphological adaptations were observed across multiple limbic and hypothalamic regions, implying a brain wide remodeling of microglial structure rather than a hippocampus restricted phenomenon (Ali et al., 2019). In the same middle age EE paradigm, Tmem119 transcript abundance also increased alongside these morphological changes, providing convergent evidence that enrichment related microglial remodeling can be detected beyond Iba1 morphology alone (Ali et al., 2019).

• Density and antigen expression

Several studies documented increased microglial density (measured as Iba1+ cell number or Iba1 immunoreactivity) following EE, particularly with prolonged exposure (Ali et al., 2019; Singhal et al., 2020a,^2021^). Broad increases in Iba1 expression were observed across hippocampal, hypothalamic, and amygdalar regions in middle-aged mice after long-term EE (Ali et al., 2019). These changes were interpreted as reflecting enhanced microglial presence or surveillance rather than overt activation.

Short-term EE paradigms also increased Iba1+ microglial numbers in specific subregions, particularly within the dentate gyrus, although these effects were less consistent across CA1 and CA3 fields and appeared sensitive to EE duration and age at onset (Singhal et al., 2020a). Importantly, increased Iba1 expression in EE exposed animals was not accompanied by parallel increases in classical inflammatory markers, supporting the interpretation that elevated antigen expression reflects cytoskeletal remodeling, enhanced branching, or increased detection sensitivity rather than inflammatory activation (Ali et al., 2019; Singhal et al., 2021). A study additionally reported region-specific dissociations between microglial number and morphology, indicating that EE can independently regulate microglial proliferation, survival, or antigen expression depending on local circuit demands (Singhal et al., 2020a).

• Activation and proinflammatory vs. homeostatic states

EE consistently promoted a shift toward a more homeostatic or neuroprotective microglial phenotype in physiological aging models (Xu et al., 2016; Ali et al., 2019). In aged or middle-aged rodents, EE reduced markers of pro-inflammatory activation (e.g., decreased CD68 or MHC-II expression) and attenuated exaggerated inflammatory responses compared to standard housed controls (Williamson et al., 2012; Xu et al., 2016). Microglia in EE-exposed animals often exhibited lower expression of pro-inflammatory genes or cytokines, maintaining a surveillance state more akin to younger animals (Williamson et al., 2012). This was particularly evident in long-term paradigms, where EE mitigated age-related priming of microglia toward a reactive state (Ali et al., 2019; Singhal et al., 2020a). This pattern is also supported by late-life enrichment studies in transgenic models, where EE reduced overall microglial burden while promoting a more functional, phagocytic phenotype without exacerbating inflammatory signaling (Stuart et al., 2019).

In late-life enrichment studies in transgenic models, EE reduced overall microglial burden while promoting a more functional, phagocytic phenotype without exacerbating inflammatory signaling (Stuart et al., 2019). In challenge-based paradigms, EE also blunted microglial hyperresponsiveness to inflammatory stimuli such as systemic lipopolysaccharide or intracerebral amyloid-β exposure, indicating that EE reduces microglial priming rather than merely suppressing baseline inflammation (Williamson et al., 2012; Xu et al., 2016). Cytokine profiling revealed downregulation of chemokines and proinflammatory mediators (including TNF family members and CC chemokines) alongside preservation of immune homeostasis-related gene expression patterns (Williamson et al., 2012). Notably, EE induced shifts favored functional phenotypes associated with tissue maintenance rather than immune suppression, suggesting qualitative remodeling of activation state rather than global suppression of microglial activity (Xu et al., 2016). Similarly, in aged mice exposed to social isolation, short-term EE reduced hippocampal pro-inflammatory markers and improved cognitive outcomes, further supporting its role in modulating neuroinflammatory tone in late life (Li et al., 2025). Recent multiomic hippocampal work further suggests that aging responsive EE effects can also be captured within transcriptionally defined microglial populations at single cell level, although these studies did not quantify Tmem119 or P2ry12 protein endpoints (Perez et al., 2024).

• Senescence reduction

Evidence on senescence markers in microglia remains limited and largely emerging. Direct quantification of classic senescence-associated proteins such as p16INK4a or p21 specifically in microglia under EE conditions was not reported in any included studies. Data characterizing p16-high microglial subsets as senescent populations are primarily derived from broader aging or neurodegenerative disease models rather than EE-specific interventions. For instance, a study in general aging contexts has identified p16-high microglia as senescent populations whose clearance can improve brain function (Ogrodnik et al., 2019). A subset of direct and indirect evidence suggest that EE may counteract age-associated microglial senescence, with reduced expression of senescence-associated β-galactosidase (SA-β-gal) or senescence-associated secretory phenotype (SASP)-related factors in enriched environments (Williamson et al., 2012; Xu et al., 2016; Ali et al., 2019; Singhal et al., 2020a). The authors of these studies observed reduced pro-inflammatory cytokine production or attenuated inflammatory responses following EE, which may indirectly reflect diminished SASP activity. However, direct assessment of SA-β-gal staining or comprehensive SASP profiling in microglia post-EE was rarely performed, and findings were inconsistent across brain regions or intervention durations. These observations imply that any potential anti-senescence effects of EE on microglia may be inferred from downstream reductions in inflammatory tone rather than from direct measurement of established senescence markers. The above studies, therefore, provide only emerging and indirect support for EE counteracting microglial senescence in aging, highlighting a critical gap in the literature regarding direct senescence marker quantification in EE exposed microglia. Future studies integrating senescence-specific reporters, transcriptomic profiling, and microglia targeted interventions will be essential to clarify the relationship between EE and microglial senescence during aging.

Together, these findings suggest that EE preserves microglial structural plasticity during aging, counteracting morphological hallmarks of microglial decline and maintaining ramified, surveillance competent phenotypes. In parallel, EE enhances microglial coverage and immunosurveillance capacity across key brain regions without inducing pathological microgliosis. Beyond structural and density related effects, EE acts as a potent modulator of age-related microglial immune tone, preserving functional responsiveness while preventing maladaptive proinflammatory amplification. Collectively, the included studies indicate that EE exerts broad beneficial effects on microglial dynamics during aging by integrating morphological preservation, enhanced surveillance, promotion of homeostatic over proinflammatory states, and potential attenuation of senescence related features in a duration- and region-dependent manner. Compared with PE-only paradigms (section 3.2.2), which more consistently suppress inflammatory signaling, EE appears to exert stronger effects on microglial structural remodeling and neuroimmune interactions, particularly in long-term paradigms.

#### 3.2.2 PE effects on microglia

Studies examining the effects of PE on microglia in aging models consistently report beneficial outcomes, including attenuation of microglial activation, shifts toward more homeostatic or reparative phenotypes, and reduction of age-associated inflammatory and senescence-like features. These effects are influenced by exercise modality (voluntary wheel running vs. forced treadmill exercise), duration, age at intervention onset, and brain region, with the hippocampus, particularly the dentate gyrus and CA1, emerging as a highly responsive site. Compared to EE, PE only induced microglial responses appear more uniform in suppressing pro-inflammatory signaling in non-pathological aging, whereas EE often produces more heterogeneous or morphology-focused outcomes (Barrientos et al., 2011; Vukovic et al., 2012; Kohman et al., 2013b; Lu et al., 2017; Chauquet et al., 2024). Across multiple species and aging paradigms, PE consistently reduced hippocampal neuroinflammatory tone even when detailed morphological endpoints were not assessed, suggesting that anti-inflammatory effects represent a core and robust outcome of exercise exposure in aging microglia (Barrientos et al., 2011; Vukovic et al., 2012; Krejcova et al., 2024). Accordingly, the synthesis below distinguishes physiological aging findings from accelerated aging, disease, and immune challenge paradigms.

• Morphology and activation

Both long-term and short-term PE paradigms modulate microglial activation states during aging, though the magnitude and cellular readouts vary. Longer PE protocols (≥8 weeks), initiated in middle-aged or aged animals, consistently attenuate age-related microgliosis and activated phenotypes, particularly within hippocampal subfields (Vukovic et al., 2012; Krejcova et al., 2024). For example, voluntary wheel running reduced activated microglial profiles and restored functional support for neural precursor cells in the aged dentate gyrus via CX3CL1-CX3CR1 dependent mechanisms (Vukovic et al., 2012). Similarly, stereological analyses further demonstrated that PE reduced age-related increases in microglial numerical density across molecular, granular, and polymorphic layers of the dentate gyrus, indicating broad suppression of microgliosis rather than focal regional effects (Krejcova et al., 2024).

Shorter PE interventions (4–8 weeks) also exert anti-inflammatory effects, though these are often detected at the level of cytokine expression or activation markers rather than detailed morphology. Voluntary wheel running for 6 weeks reduced hippocampal IL-1β and IL-6 levels and increased the anti-inflammatory cytokine IL-1RA in aged animals, indicating suppression of a pro-inflammatory milieu despite the absence of formal morphometric analysis (Barrientos et al., 2011). In metabolic and accelerated aging models, PE shifted microglia from hypertrophic, amoeboid morphologies toward more ramified, resting-like profiles, highlighting the capacity of exercise to reverse pathology associated structural activation (Yoo et al., 2015; Liu et al., 2024).

Across models, PE consistently suppressed pro-inflammatory activation markers (e.g., iNOS, CD86, CD32) while enhancing reparative or reparative associated markers (e.g., ARG1, CD206, TGF-β), particularly in hippocampal CA1 and dentate gyrus regions (Lu et al., 2017; Rodriguez et al., 2015). This shift in marker expression was especially prominent in disease or immune challenged contexts, where PE reduced cytokine burden and promoted tissue repair associated microglial states (Yoo et al., 2015; Lu et al., 2017).

Importantly, sex- and region-dependent effects were noted, with aged females often showing more pronounced reductions in hippocampal microglial activation following voluntary running than males (Kohman et al., 2013b). In immune-challenged aging models, PE blunted exaggerated microglial responses to systemic inflammatory stimuli, preserving a surveillance oriented rather than reactive phenotype (Kohman et al., 2013b; Yoo et al., 2015). These findings indicate that PE reduces age-related microglial priming, thereby limiting exaggerated inflammatory reactivity to secondary immune insults.

• Density and antigen expression

Physical exercise modulates microglial density and antigen expression in aging models, often in a duration- and region-specific manner. Longer interventions (≥8 weeks) typically reduce age-associated increases in microglial numerical density (Nv, #/mm^3^), particularly in hippocampal subfields (Krejcova et al., 2024). For instance, layer-resolved stereology showed that age-related microglial accumulation occurs across molecular, granular, and polymorphic layers, and late-life treadmill exercise partially normalized this laminar distribution rather than producing uniform reductions across the dentate gyrus (Krejcova et al., 2024).

In accelerated-aging and AD-related models, aerobic treadmill exercise reduced Iba1+ cell density and Iba1 optical density in hippocampus and concurrently suppressed inflammatory pathway signaling (RIPK1–NF-κB and MAP3K5/JNK), supporting the interpretation that reduced antigen load accompanies reduced inflammatory activation rather than reflecting a purely technical staining effect (Liu et al., 2024). PE-related changes in microglia density can be more complex. For example, in 3xTg-AD mice, long-term running alone promoted microglial hypertrophy in CA1 (increased microglial surface/volume and soma volume), whereas combined EE+PE prevented AD associated increases in microglial numerical density (Nv) in CA1 strata, indicating that exercise can alter microglial burden via either density or cell-size/domain changes (Rodriguez et al., 2015).

In physiological aging, short-term PE (4–8 weeks) shows more variable effects on density but consistently downregulates pro-inflammatory antigens like MHC II and CD86 (Kohman et al., 2012, 2013b). Flow cytometry specifically demonstrated that aging increased the proportion of activated hippocampal microglia (MHC II+ and CD86+), and voluntary running reduced these activated fractions in aged females, while effects were smaller or absent in males and differed by region when comparing hippocampus versus whole brain preparations (Kohman et al., 2013b). Consistent with this, running shifted microglia toward a less activated profile in the aged dentate gyrus by reducing the proportion of MHC II+ microglia and restoring CX3CL1-CX3CR1 signaling, indicating that antigen expression changes can reflect altered functional state rather than changes in cell number (Vukovic et al., 2012). In immune-challenged contexts, PE prevented LPS-induced upregulation of activation markers (CD68, iNOS), maintaining lower antigen load (Mela et al., 2020). A recent aged female single cell study similarly found that voluntary exercise rejuvenated hippocampal microglial transcriptional states within Tmem119 annotated populations while reducing brain T-cell accumulation, extending the PE literature beyond Iba1-only readouts (Chauquet et al., 2024).

Taken together, PE attenuates age-related increases in inflammatory antigen expression (e.g., decrease in the expression of CD11b, CD68), promoting a less primed profile, though density reductions are more evident in hippocampal than cortical regions.

• Activation and proinflammatory vs. homeostatic states

In non-pathological aging, PE consistently attenuates age-related microglial activation, shifting microglial profiles away from pro-inflammatory states to more homeostatic or reparative states (Lu et al., 2017; Mela et al., 2020; Chauquet et al., 2024). In longer PE paradigms, this corresponded to reduced pro-inflammatory markers (iNOS, CD86) and enhances reparative associated expression (CD206, Arg1), decreasing cytokines like IL-1β, TNF-α, IL-6 (Mela et al., 2020; Chauquet et al., 2024). Cytokine-focused aging studies further show that even moderate voluntary running reduces hippocampal IL-1β and IL-6 while increasing IL-1RA in aged animals, consistent with a broad anti-inflammatory shift in the aged hippocampal milieu (Barrientos et al., 2011). Moreover, exercise reduced inflammatory sensitivity of microglia isolated from aged brains (*ex vivo*), supporting the interpretation that PE suppresses microglial priming rather than only lowering bulk tissue cytokine levels (Barrientos et al., 2011).

In immune-challenge paradigms, short-term PE blunts secondary inflammatory responses (e.g., LPS), preserving homeostatic surveillance (Barrientos et al., 2011; Kohman et al., 2013b; Littlefield et al., 2015). For instance, in the LPS challenge paradigm with aged mice, voluntary exercise protected against inflammation induced functional decline and maintained proneurogenic microglial features (increased BDNF+ microglia), consistent with preservation of a supportive/homeostatic phenotype under immune stress (Littlefield et al., 2015).

In pathology models, PE suppresses NF-κB/JNK signaling, reducing pro-inflammatory activation (Lu et al., 2017; Liu et al., 2024). For example, in STZ induced AD-like pathology, treadmill exercise suppressed pro-inflammatory microglial markers (CD32, CD86, iNOS) and increased reparative associated proteins (ARG1, TGF-β, CD206), alongside increases in anti-inflammatory cytokines (IL-4, IL-10) and reductions in IL-1β and TNF-α, indicating a coordinated remodeling of microglial state rather than selective marker suppression (Lu et al., 2017). Similarly, in accelerated inflammaging, exercise suppressed RIPK1-linked NF-κB activation and MAP3K5/JNK signaling, aligning pathway level inhibition with reduced microglial activation readouts and improved neuronal viability (Liu et al., 2024).

Sex differences have also been noted with females showing stronger anti-inflammatory shifts. Specifically, running reduced basal activation marker expression (MHC II and CD86) in aged females hippocampal microglia, while CD206 remained low overall, suggesting that in some non-pathological aging contexts PE primarily reduces proinflammatory activation rather than inducing a uniform reparative microglial profile, particularly in females (Kohman et al., 2013b).

Mechanistically, exercise induced shifts toward homeostatic microglial states have been linked to restoration of CX3CL1 levels and CX3CL1-CX3CR1 signaling in the aged dentate gyrus, relieving microglia mediated suppression of neural precursor cell activity and supporting neurogenesis (Vukovic et al., 2012). At the transcriptional level, single-cell profiling showed that voluntary running reversed a large fraction of age-associated microglial inflammatory/DAM-like gene programs in hippocampus, restoring a young-like homeostatic signature and reducing immune-activated clusters (Chauquet et al., 2024).

• Senescence reduction

A subset of studies directly implicates PE in reducing microglial senescence-like features during aging. In aged sedentary animals, microglia exhibit increased expression of senescence-associated markers, including p16^INK4A^ and β-galactosidase, alongside elevated inflammatory output (Kohman et al., 2012). Short-term treadmill exercise significantly reduced these markers, indicating partial reversal of senescence-associated phenotypes (Kohman et al., 2012). Direct cellular analyses confirmed that PE reduced the proportion of p16INK4A^+^ and SA-β-gal^+^ microglia, providing some of the direct evidence that exercise attenuates microglial senescence rather than merely suppressing inflammation (Mela et al., 2020).

Transcriptomic analyses further revealed that PE reverses aging associated microglial gene signatures enriched for immune activation, interferon signaling, and disease-associated microglia like states, restoring a more youthful, homeostatic transcriptional profile (Kohman et al., 2013a). Single cell RNA sequencing demonstrated that voluntary running reversed up to ∼90% of aging associated differentially expressed genes in hippocampal microglia, markedly reducing DAM-like and interferon responsive clusters (Chauquet et al., 2024).

Importantly, these anti-senescent effects do not necessarily require lifelong intervention. Even relatively brief PE paradigms were sufficient to blunt age-related priming and exaggerated inflammatory responsiveness, particularly in hippocampal microglia (Kohman et al., 2012). While classical senescence markers were not assessed in all studies, reductions in inflammatory bias, restoration of neurotrophic signaling (e.g., BDNF-associated microglial subsets), and suppression of DAM-like transcriptional programs collectively support an exercise-induced delay or reversal of functional microglial aging (Kohman et al., 2013a). Together, these findings position PE as a powerful modulator of microglial aging, capable of simultaneously restoring metabolic function, suppressing senescence-associated programs, and re-establishing homeostatic immune surveillance even when initiated later in life. In contrast to EE, which primarily influences morphology and immune interface dynamics, PE effects are more consistently reflected in suppression of pro-inflammatory signaling and reversal of age-related transcriptional activation states.

#### 3.2.3 Direct EE vs. PE comparison on microglia

Synthesizing the EE and PE evidence presented above, it is evident that both interventions promote a more homeostatic, ramified microglial phenotype in aged or middle-aged rodents and attenuate pro-inflammatory markers such as CD68, MHC-II, IL-1β and TNF-α. The interventions differ, however, in mechanism and in the dimension on which the effect is most pronounced. EE more consistently increases microglial coverage and process complexity in limbic and hypothalamic regions over long durations and modulates neuroimmune interactions including the response to peripheral immune or amyloid challenge. PE more consistently reverses age related transcriptional priming, including downregulation of inflammatory and MHC-II programs and partial restoration of homeostatic gene signatures, with stronger effects on hippocampal neurogenesis coupled microglial states. Where head-to-head data exist, combined EE+PE does not consistently outperform either modality alone for microglial outcomes. The structural surveillance bias of EE and the metabolic/transcriptional bias of PE are best read as complementary rather than redundant effects on aged microglia.

### 3.3 Astrocytes

Aged astrocytes show enlarged soma, thickened processes, and reduced territorial coverage, accompanied by upregulation of complement- and inflammation-associated reactive genes that are most pronounced in hippocampus and striatum (Clarke et al., 2018; Ashraf et al., 2019). This transcriptional shift is driven, at least in part, by microglia derived IL-1, TNF, and C1q, and is accompanied by impaired glycogen and lactate metabolism, mitochondrial dysfunction, and reduced expression of neurotrophic and neuroprotective factors including BDNF, VEGF, FGF-2 and humanin (Bernal and Peterson, 2011; Zarate et al., 2019; Hanslik et al., 2021; Sikora et al., 2021). EE-related astrocytic outcomes are interpreted against this baseline of reactive transcriptional reprogramming and metabolic decline.

#### 3.3.1 EE effects on astrocytes

The included studies examining the effects of EE on astrocytes in aging models reported heterogeneous but generally beneficial outcomes, with changes in astrocytic morphology, density, GFAP expression, and reactivity states. Effects appeared to vary by intervention duration, brain region, and whether EE paradigms included locomotor components. Compared to microglia, astrocytic responses to EE were less uniformly consistent across studies, with some evidence suggesting EE-induced increases in GFAP immunoreactivity or astrocytic complexity that could superficially resemble age-related hypertrophy, but were often interpreted as enhanced supportive or plastic capacity rather than pathological reactivity (Diniz et al., 2010, 2016; Sampedro-Piquero et al., 2014; Bento-Torres et al., 2017). Importantly, this heterogeneity reflects the high degree of regional specialization and functional diversity of astrocytes, which may confer differential sensitivity to environmental stimuli across hippocampal layers, cortical regions, and pathological contexts (Diniz et al., 2010, 2016; Sampedro-Piquero et al., 2014). Across studies, astrocytic outcomes under EE were shaped by exposure duration and life stage at intervention onset, with long-term or lifelong EE producing more consistent remodeling than short-term paradigms (Diniz et al., 2010, 2016; Singhal et al., 2020a). Where relevant, physiological aging findings are separated below from disease, neurodegenerative, and injury/inflammatory paradigms.

• Morphology and hypertrophy

Long-term EE (typically ≥6 months, initiated in young adulthood or middle age) frequently increased astrocytic process complexity and ramification in regions such as the hippocampus (particularly dentate gyrus molecular layer) and cortex. For instance, EE promoted greater morphological diversity or convergence toward a more elaborate astrocytic phenotype compared to age-matched standard-housed controls, as assessed by GFAP immunohistochemistry and Sholl analysis or process tracing (Diniz et al., 2010; Sampedro-Piquero et al., 2014). Three-dimensional reconstructions further revealed increases in branch length, intersection number, and territorial coverage, indicating enhanced astrocytic domain organization rather than diffuse hypertrophy (Diniz et al., 2016).

In contrast, short-term EE (4–12 weeks) produced more modest or region-specific remodeling, sometimes limited to increased GFAP+ process extension in hippocampal subfields (Singhal et al., 2020a). Several studies noted that these short-term effects were insufficient to restore age-related losses in astrocytic morphological diversity, suggesting a temporal threshold for meaningful astroglial remodeling (Singhal et al., 2020a,^2021^).

Paradoxically, some reports noted that EE-induced astrocytic changes in young animals resembled age-related hypertrophy (e.g., increased GFAP expression and process thickness), but in aging contexts, these shifts were linked to preserved supportive functions rather than maladaptive reactivity (Soffie et al., 1999; Williamson et al., 2012). Notably, lifelong EE prevented the age-related collapse of complex astrocyte morphotypes (Type I phenotypes), maintaining heterogeneous astrocytic populations associated with synaptic support and plasticity (Diniz et al., 2016).

In physiological aging models, EE appeared to counteract age-related simplification or atrophy of astrocytic arbors in select paradigms, potentially supporting synaptic maintenance and glutamate homeostasis (Diniz et al., 2016; Bento-Torres et al., 2017). In disease, neurodegenerative, or injury-related models (e.g., stroke, amyloid models), EE reduced proliferating astrocytes and glial scar volume, further mitigating hypertrophic responses (Buchhold et al., 2007; Beauquis et al., 2013). These findings suggest that EE promotes adaptive hypertrophy in normative aging while limiting excessive or maladaptive astrocytic enlargement under pathological stress.

• Density and GFAP expression

Environmental enrichment consistently increased the number or density of GFAP-immunoreactive astrocytes in the hippocampus (molecular layer) and other limbic regions, particularly with prolonged exposure starting earlier in life (Diniz et al., 2010; Singhal et al., 2020a). Layer-specific analyses revealed that EE effects were most pronounced in regions with high synaptic turnover, such as the dentate gyrus molecular layer, whereas granular and polymorphic layers were less consistently affected (Diniz et al., 2010; Cunha Feio, Leal et al., 2023). This elevation was often interpreted as adaptive plasticity enhancing astrocytic coverage and support for neuronal networks, rather than reactive gliosis (Williamson et al., 2012; Sampedro-Piquero et al., 2014). In middle-aged rodents, duration-dependent effects were evident. Longer EE protocols (e.g., 6–12 months) produced more robust increases in astrocyte number compared to shorter interventions (Singhal et al., 2020a,^2021^). Conversely, lifelong EE prevented age-related increases in astrocyte density observed under standard housing, suggesting that EE constrains excessive astroglial accumulation linked to aging (Soffie et al., 1999).

Few studies separated cognitive/sensory EE components from physical activity (e.g., running wheels), but those that did suggested the astrocytic density increase might be partially attributable to locomotor elements (Williamson et al., 2012). In aged animals, EE increased astrocyte numbers in specific layers (e.g., molecular layer of dentate gyrus), but masticatory or environmental factors modulated responses in stratum radiatum (Cunha Feio, Leal et al., 2023). Together, these findings indicate that EE fine tunes astrocyte density in a region-, layer-, and duration-dependent manner rather than uniformly increasing astrocyte numbers across the brain.

• Reactivity and functional state shifts

In disease, neurodegenerative, or injury related models, EE generally attenuated age-related astrocytic reactivity, reducing hypertrophic or dystrophic features and proinflammatory markers (e.g., lower vimentin co-expression or attenuated cytokine release) (Buchhold et al., 2007; Beauquis et al., 2013). In amyloid-associated contexts, EE normalized aberrant plaque-associated astrocyte morphologies and prevented early pathological remodeling in non-plaque-associated astrocytes, yielding a more control-like astroglial phenotype (Beauquis et al., 2013).

In non-pathological aging, EE promoted a shift toward a more homeostatic or neuroprotective astrocytic phenotype, potentially via enhanced neurotrophic support (e.g., BDNF expression in astrocytes) and reduced inflammatory tone (Williamson et al., 2012; Sampedro-Piquero et al., 2014). However, direct state defining marker panels were rarely assessed in pure aging + EE studies. As a result, conclusions regarding astrocytic polarization remain largely inferential and based on functional readouts rather than binary molecular classification. In contrast to microglia, where EE modulated neuroimmune interfaces, astrocytic effects under EE appeared more tied to structural remodeling and supportive functions (e.g., glutamate uptake, metabolic shuttling) (Diniz et al., 2010; Bento-Torres et al., 2017). In inflammatory challenge paradigms, EE also blunted LPS-induced reactivity, particularly in the dentate gyrus, suggesting anti-inflammatory modulation (Williamson et al., 2012). Together with the disease and injury findings above, these observations support the role for EE in dampening excessive astrocytic inflammatory responsiveness while preserving adaptive reactivity outside normative aging contexts.

• Supportive functions

Limited but emerging evidence linked EE-induced astrocytic changes to improved neuronal support, including enhanced metabolic shuttling (lactate supply to neurons), better synaptic coverage, and facilitation of neurogenesis or synaptic plasticity in aged animals (Beauquis et al., 2013; Diniz et al., 2016). Astrocytic morphological complexity under EE was consistently associated with improved hippocampal dependent memory performance, suggesting functional relevance of structural remodeling (Sampedro-Piquero et al., 2014; Diniz et al., 2016). These supportive roles were proposed as mediators of EE’s cognitive benefits in aging, though causal links remain correlational (Sampedro-Piquero et al., 2014; Cunha Feio, Leal et al., 2023). EE preserved diversity in astrocyte phenotypes (e.g., Type I high-complexity vs. Type II low-complexity), which may underpin resilience against age-related memory impairments (Diniz et al., 2016). Together, these observations implicate astrocytes as key intermediaries linking environmental stimulation to sustained synaptic and metabolic support during aging.

Collectively, the included studies suggest that EE exerts region- and duration-dependent effects on astrocytic dynamics during aging, primarily by increasing density and morphological complexity (potentially adaptive hypertrophy), attenuating pathological reactivity, and preserving or enhancing supportive functions. Compared with microglia, astrocytic responses to EE appear more variable and slower to emerge, requiring longer exposure durations and exhibiting greater dependence on regional context and experimental design.

#### 3.3.2 PE effects on astrocytes

The studies examining the effects of PE on astrocytes in aging and aging-related models reported heterogeneous but generally beneficial outcomes, with changes in astrocytic morphology, density, GFAP expression, and reactivity states. Effects appeared to vary by intervention duration, brain region, and whether paradigms included voluntary or forced components. Compared to microglia, astrocytic responses to PE were less uniformly consistent across studies, with evidence suggesting PE induced increases in GFAP immunoreactivity or astrocytic complexity resemble age related hypertrophy and often interpreted as enhanced supportive or plastic capacity rather than pathological reactivity (Latimer et al., 2011; Tapia-Rojas et al., 2016; Belaya et al., 2020; Yamaguchi et al., 2023). PE can both increase and decrease GFAP defined astrocytic reactivity depending on disease context, brain region, and exercise modality, underscoring that astrocytic responses to PE are bidirectional rather than uniformly suppressive (Latimer et al., 2011; Tapia-Rojas et al., 2016; He et al., 2017; Belaya et al., 2020). Because much of the PE astrocyte literature arises from non-physiological models, the specific model context is identified explicitly below.

• Morphology and hypertrophy

Long-term PE (typically ≥6 months, initiated in young adulthood or middle age) frequently increased astrocytic process complexity and ramification in regions such as the hippocampus (particularly dentate gyrus molecular layer) and cortex. For instance, PE promoted greater morphological diversity or convergence toward a more elaborate astrocytic phenotype compared to age matched standard housed controls, as assessed by GFAP immunohistochemistry and Sholl analysis (Belaya et al., 2020).

In contrast, short-term PE (4–8 weeks) produced more modest or region-specific remodeling, sometimes limited to increased GFAP+ process extension in hippocampal subfields (Latimer et al., 2011). Short-duration interventions also revealed circuit-specific effects, with astrocytic hypertrophy observed in hippocampal CA1 or dentate gyrus but not uniformly across cortex or striatum (Latimer et al., 2011). In physiological aging paradigms, PE therefore appeared to counteract age related simplification or atrophy of astrocytic arbors in select paradigms, potentially supporting synaptic maintenance and glutamate homeostasis (Tapia-Rojas et al., 2016; Yamaguchi et al., 2023). In stress and disease models (e.g., chronic unpredictable stress, alcohol exposure, AD), PE restored astrocytic morphological complexity and normalized soma size and process overgrowth, reversing pathology induced simplification or excessive hypertrophy rather than producing uniform enlargement (Liu et al., 2023, 2024; Li et al., 2024). Notably, some PE paradigms induced astrocytic hypertrophy selectively in plaque associated astrocytes while sparing distant astrocytes, suggesting spatially restricted remodeling linked to local pathology rather than global astrogliosis (Belaya et al., 2020).

• Density and GFAP expression

In physiological aging, PE consistently increased the number or density of GFAP immunoreactive astrocytes in the hippocampus (molecular layer) and other limbic regions, particularly with prolonged exposure starting earlier in life (Belaya et al., 2020). Several studies, however, demonstrated astrocytic hypertrophy without changes in total astrocyte number, indicating that PE-induced increases in GFAP signal often reflect structural remodeling rather than astrocyte proliferation (Latimer et al., 2011). In middle-aged rodents, duration dependent effects were evident: longer PE protocols (e.g., 6–12 months) produced more robust increases in astrocyte number compared to shorter interventions (Latimer et al., 2011). Conversely, in aging and Alzheimer’s disease models, voluntary running reduced astrocyte number and GFAP immunoreactivity in hippocampus, cortex, or medial prefrontal cortex, indicating suppression of pathological astrocyte hyperplasia rather than loss of supportive astrocytes (Tapia-Rojas et al., 2016; He et al., 2017; Liu et al., 2023). In aged animals, PE increased astrocyte numbers in specific layers (e.g., molecular layer of dentate gyrus), but factors like stroke modulated responses in peri-infarct regions (Yamaguchi et al., 2023). Following ischemic injury, PE did not increase total astrocyte number but selectively increased the proportion of newborn GFAP^+^ astrocytes, indicating phenotypic remodeling rather than density increase (Yamaguchi et al., 2023).

• Reactivity and functional state shifts

In disease, injury, and accelerated-aging models, PE generally attenuated astrocytic reactivity, reducing hypertrophic or dystrophic features and proinflammatory markers (e.g., lower vimentin co-expression or attenuated cytokine release) (Tapia-Rojas et al., 2016; Yamaguchi et al., 2023). In Alzheimer’s disease models, voluntary running reduced astrogliosis as measured by GFAP intensity, astrocyte soma size, and S100B expression, even when astrocytic hypertrophy or C3 expression was not fully reversed (Tapia-Rojas et al., 2016; Liu et al., 2023). In non-pathological aging, PE promoted a shift toward a more homeostatic or neuroprotective astrocytic phenotype, potentially via enhanced neurotrophic support (e.g., BDNF expression in astrocytes) and reduced inflammatory tone (Belaya et al., 2020). Exercise induced astrocytic activation was frequently characterized as protective reactivity, with increased S100A10^+^/GFAP^+^ astrocytes and reduced C3^+^/GFAP^+^ astrocytes in hypoperfusion and ischemia models (Chen et al., 2021; Cao et al., 2022).

However, direct state defining marker panels were rarely assessed in pure aging + PE studies. Where assessed, PE suppressed neurotoxic astrocytic signaling pathways (e.g., ERK/JNK, Lcn2 expression, STAT3-dependent maladaptive signaling) while promoting synapse supportive programs (Chen et al., 2021; Cao et al., 2022; Yamaguchi et al., 2023). In contrast to microglia, where PE modulated neuroimmune interfaces, astrocytic effects under PE appeared more tied to structural remodeling and supportive functions (e.g., glutamate uptake, metabolic shuttling) (Belaya et al., 2020).

• Supportive functions

Limited but emerging evidence linked PE induced astrocytic changes to improved neuronal support, including enhanced metabolic shuttling (lactate supply to neurons), better synaptic coverage, and facilitation of neurogenesis or synaptic plasticity in aged animals (Belaya et al., 2020). Multiple studies directly demonstrated enhanced astrocyte-synapse structural coupling following PE, including increased PSD95^+^ puncta contacting astrocytic processes in hippocampus, prefrontal cortex, and motor cortex (Belaya et al., 2020; Liu et al., 2023; Li et al., 2024). These supportive roles were proposed as mediators of PE’s cognitive benefits in aging, though causal links remain correlational (Latimer et al., 2011). More mechanistic evidence showed that PE restores astrocytic mitochondrial health and promotes CD38-dependent mitochondrial transfer from astrocytes to neurons, directly reducing neuronal oxidative stress and improving cognition in AD models (Cai et al., 2025). PE also restored astrocytic AQP4 polarity at perivascular end feet, enhancing glymphatic clearance of amyloid-β and indirectly supporting synaptic integrity in aged animals (He et al., 2017). In injury and stress models, PE induced astrocytic STAT3–Gpc6 signaling, GLT-1 upregulation, and normalization of astrocytic calcium dynamics were causally linked to synaptogenesis, dendritic spine stabilization, and functional recovery (Chen et al., 2021; Li et al., 2024; Liu et al., 2024).

Collectively, PE exerts region and duration dependent effects on astrocytic dynamics during aging, primarily by remodeling astrocytic morphology, normalizing pathological GFAP expression and reactivity, and enhancing astrocyte mediated supportive functions. These changes may contribute to PE’s neuroprotective role, though effects appear more context dependent and mechanistically diverse than those observed in microglia, and often reflect qualitative remodeling of astrocytic function rather than uniform suppression or expansion of astrocyte populations.

#### 3.3.3 Direct EE vs. PE comparison on astrocytes

Both EE and PE reduce GFAP positive astrocyte density and reactive morphology in age-related and aging relevant models, preserve astrocyte process complexity and territorial coverage, and partially restore expression of neurotrophic and metabolic support genes. EE shows particularly clear effects on hippocampal and cortical astrocyte morphology under long duration paradigms, and modulates astrocyte microglia crosstalk via reduced microglia derived IL-1, TNF and C1q. PE shows more consistent effects on astrocyte metabolism (glycogen lactate coupling, mitochondrial function) and on BDNF/VEGF/FGF-2 related neurotrophic support, with region specific remodeling that often parallels exercise induced vascular and neurogenic changes. Combined EE+PE generally produces effects comparable to the stronger single modality rather than additive benefits on astrocytic endpoints. Taken together, EE preferentially shapes astrocyte structural and immune coupled phenotypes, whereas PE preferentially shapes astrocyte metabolic and neurotrophic phenotypes.

### 3.4 Oligodendrocytes

Oligodendrocyte aging is characterized by reduced proportions of mature MBP positive cells, accumulation of senescence markers (CDKN1A, CDKN2A) and integrated stress response signaling (phospho-eIF2α), and calpain1 mediated degradation of myelin proteins including myelin associated glycoprotein, CNPase and MOSP (Sloane et al., 2003; Schlett et al., 2023; Windener et al., 2024). Remyelinating internodes formed in aged animals are shorter and thinner than the originals, slowing conduction, and OPC differentiation capacity is reduced (Peters, 2009; Sams, 2021). Periventricular and frontal white matter are particularly vulnerable, and CD8+ T-cell derived interferon signaling promotes IFN responsive oligodendrocyte states in aged white matter (Simpson et al., 2007; Miyamoto et al., 2013; Kaya et al., 2022). The APOE4 genotype additionally impairs oligodendrocyte function independent of amyloid pathology (Cheng et al., 2022). PE related oligodendrocytes and myelin outcomes are interpreted within this framework.

Only a few studies directly examined the effects of EE or PE on oligodendrocytes in aging and aging-related models. Available evidence on oligodendrocytes lineage cells (mature oligodendrocytes and oligodendrocyte precursor cells, OPCs) remains limited and heterogeneous, with most data derived from young, adult or disease models rather than pure aging contexts (Pusic and Kraig, 2014; Jensen et al., 2018; Maugeri et al., 2023). Where reported, EE and PE appear to exert modest, context dependent effects on myelin integrity, OPC proliferation, or remyelination capacity, but these are not consistently superior to controls in aged animals (Chen et al., 2020; Graciani et al., 2023).

• Oligodendrocytes and myelin

In aging rodents, age related myelin loss, reduced OPC proliferation, and impaired remyelination capacity contribute to white matter atrophy and slowed neural conduction. Long-term EE (started at young or middle age) has been associated with preservation or modest enhancement of myelin integrity and OPC dynamics in select hippocampal and cortical regions, potentially through activity dependent mechanisms (e.g., increased BDNF or IGF-1 signaling) (Pusic and Kraig, 2014). However, direct comparisons in aged models are scarce, and effects are often confounded by running wheels in EE paradigms. PE (voluntary wheel running) shows more consistent, albeit modest, benefits in delaying age-related demyelination and supporting OPC proliferation or survival in white matter tracts (Jensen et al., 2018; Chen et al., 2020; Graciani et al., 2023; Maugeri et al., 2023). Combined EE+PE interventions have not been systematically compared for oligodendrocytes outcomes in aging, and no additive effects have been clearly demonstrated. No studies reported significant changes in oligodendrocyte number, morphology, or marker expression (e.g., MBP, PLP, NG2) that were uniquely attributable to EE or PE in non-pathological aged brain.

### 3.5 Direct or indirect comparisons between environmental enrichment and physical exercise

This section synthesizes evidence from studies that directly or indirectly compare the effects of EE and PE on glial cell dynamics during aging. Direct comparisons are limited to a small number of preclinical studies that included both interventions in the same experimental design, often with combined EE+PE arms. These include one physiological aging study and one amyloid-beta neurotoxicity model. Indirect comparisons are interpreted with explicit attention to whether the underlying evidence comes from normative aging, disease/neurodegenerative, or stress/injury paradigms.

#### 3.5.1 Direct comparison of EE and PE

Only a few studies directly compared EE, PE, and/or their combination in aging models with glial specific outcomes. A small number of preclinical studies provide head-to-head insights, primarily focusing on middle-aged or aged mice and emphasizing microglial and astrocytic endpoints (Prado Lima et al., 2018; Singhal et al., 2021).

In one study of C57BL/6 mice starting at 3, 8, or 13 months of age, short-term EE (7 weeks) was compared to PE (voluntary wheel running) and combined EE+PE (Singhal et al., 2021). EE alone increased the number of Iba1+ microglia in the dentate gyrus at 5 and 10 months, while PE and PE+EE increased it only at 10 months. No significant changes in GFAP+ astrocyte numbers were observed in any treatment group compared to controls. All treatments induced differences in peripheral T cell subsets from cervical lymph nodes. For example, EE increased CD8+ naïve T cell proportions at 15 months of age, and PE+EE increased CD4+ and CD8+ naïve T cell proportions at 5 months of age, but no overall changes in total CD4+ or CD8+ proportions were noted. PE showed weaker effects on microglia compared to EE, while combined EE+PE yielded non additive effects to single interventions for glial outcomes. The study highlighted EE’s role in modulating glial immune interfaces, potentially without additive benefits from combining with PE.

Another head-to-head study in an amyloid beta neurotoxicity model (young adult rats, but with aging relevant neuroinflammation) compared EE, PE (treadmill running), and social enrichment (Prado Lima et al., 2018). EE and PE both reduced microglial activation (e.g., decreased CD68+ and Iba1+ markers) and astrogliosis (e.g., lower GFAP intensity) in the hippocampus, with additive benefits from combined EE+PE on memory and neurogenesis but non superior effects on glial modulation compared to single interventions. EE showed modulation of neuroimmune crosstalk (e.g., reduced proinflammatory cytokines like IL-1β), while PE emphasized anti-inflammatory remodeling (e.g., via BDNF-TrkB signaling). However, this study’s focus on disease models limits direct applicability to normal aging.

Overall, direct head-to-head comparisons between EE and PE in normal aging models remain sparse, heterogeneous, and primarily from middle-aged rodent models. No clinical studies were identified. Combined EE+PE often produced variable or non-additive glial effects, with EE generally outperforming PE in modulating glial-immune interfaces and PE showing advantages in metabolic remodeling. Methodological differences, such as EE paradigms including running wheels, confounded pure comparisons. Evidence from amyloid, injury, and inflammatory paradigms is therefore interpreted here as complementary aging relevant context rather than direct evidence of normative aging.

#### 3.5.2 Indirect comparisons

Indirect comparisons reveal distinct yet partially overlapping effects of EE and PE on glial aging, with EE showing greater magnitude and consistency in modulating microglial morphology and neuroimmune interactions, while PE more reliably induces astrocytic remodeling and anti-inflammatory gene expression changes. Microglial comparisons are supported more strongly by physiological aging studies, whereas several astrocyte and mechanistic PE findings derive from disease, accelerated aging, or injury/stress paradigms and should be interpreted in that context. These differences vary by brain region, intervention timing, and duration, with limited data on oligodendrocytes precluding robust comparisons.

• Magnitude and consistency

Environmental enrichment consistently produced larger magnitude effects on microglial outcomes, such as increased density, ramification, and complexity (Ali et al., 2019; de Oliveira et al., 2020), with robust shifts toward homeostatic phenotypes and modulation of peripheral T-cells (Williamson et al., 2012; Singhal et al., 2021). Effects were observed often after long-term EE exposure. In contrast, PE showed moderate, more variable effects on microglia, such as reduced activation and gene expression (Kohman et al., 2012; Littlefield et al., 2015; Rodriguez et al., 2015; Mela et al., 2020; Liu et al., 2024), with consistency in reversing age-related proinflammatory states, such as downregulated NF-κB, RIPK1, and JNK pathways (Liu et al., 2024) but less impact on density or morphology.

For astrocytes, PE demonstrated greater magnitude in inducing hypertrophy and supportive remodeling, such as increased process complexity and GFAP+ territorial coverage (Latimer et al., 2011; Saur et al., 2014; Littlefield et al., 2015; Tapia-Rojas et al., 2016; Fahimi et al., 2017; He et al., 2017; Stevenson et al., 2018; Lundquist et al., 2019; Belaya et al., 2020; Chen et al., 2021; Cao et al., 2022; Kelty et al., 2022; Liu et al., 2023, 2024; Yamaguchi et al., 2023; Li et al., 2024; Cai et al., 2025), interpreted as adaptive plasticity enhancing synaptic support and glymphatic clearance (He et al., 2017; Liu et al., 2023). EE effects on astrocytes were consistent but smaller, primarily reducing pathological reactivity in disease models, such as lower GFAP expression (Soffie et al., 1999; Diniz et al., 2010, 2016; Beauquis et al., 2013; Sampedro-Piquero et al., 2014; Bento-Torres et al., 2017), without strong evidence of structural enhancement in normal aging.

Oligodendrocytes effects were inconsistently reported. PE showed modest, more consistent preservation of myelin integrity, such as delayed demyelination in white matter (Chen et al., 2020; Graciani et al., 2023; Butt et al., 2024), while the benefits of EE were subtler and context dependent, such as OPC proliferation in stimulated regions (Pusic and Kraig, 2014; Forbes and Gallo, 2017; Goldstein et al., 2021; Gao et al., 2022; Nicholson et al., 2022). Combined interventions yielded non superior glial effects compared to single modalities, with additive benefits limited to neurogenesis (Prado Lima et al., 2018; Singhal et al., 2021).

• Brain region specificity

Glial responses exhibited regional specificity, with the hippocampus (particularly dentate gyrus) showing the most consistent modulation across interventions. EE effects on microglia were widespread [hippocampus, hypothalamus, amygdala (Ali et al., 2019; Stuart et al., 2019; de Oliveira et al., 2020)], but strongest in limbic areas for neuroimmune changes (Williamson et al., 2012; Singhal et al., 2021). PE’s microglial reductions were often hippocampus specific, with variable effects in cortex or white matter (Barrientos et al., 2011; Kohman et al., 2012; Vukovic et al., 2012; Lu et al., 2017; Chauquet et al., 2024; Krejcova et al., 2024). Astrocytic changes under PE were prominent in hippocampal subfields [e.g., CA1, dentate gyrus (Saur et al., 2014; Fahimi et al., 2017; Belaya et al., 2020; Li et al., 2024)] and prefrontal/motor cortex (Lundquist et al., 2019; Liu et al., 2023, 2024; Li et al., 2024), linked to synaptic and glymphatic functions (He et al., 2017). EE’s astrocytic effects were mainly hippocampal, such as reduced gliosis in dentate gyrus (Diniz et al., 2010, 2016; Beauquis et al., 2013; Bento-Torres et al., 2017) with limited cortical data. For oligodendrocytes, PE favored white matter tracts, such as preserved integrity in corpus callosum (Chen et al., 2020; Cao et al., 2022; Graciani et al., 2023; Butt et al., 2024), while EE showed subtler effects in stimulated regions (Pusic and Kraig, 2014). Regional patterns suggest EE’s broader impact on experience dependent areas and PE on activity driven metabolic hubs, though few studies examined multiple regions simultaneously.

• Age at intervention onset

Effects of EE and PE on glial cells varied by lifespan stage, with more consistent benefits observed when interventions were initiated in young adulthood or middle age compared to late life. For microglia, EE initiated in young (e.g., 3–5 months) or middle-aged rodents (e.g., 8–10 months) increased density and shifted toward homeostatic phenotypes in the hippocampus (Kohman et al., 2012; Ali et al., 2019; de Oliveira et al., 2020; Singhal et al., 2021), but effects diminished in aged models (e.g., >18 months), where only modest reductions in activation were noted (Stuart et al., 2019; Yan et al., 2023). PE showed similar age dependence, with anti-inflammatory remodeling (e.g., reduced IL-1β, TNF-α) more pronounced in middle-aged animals (Barrientos et al., 2011; Kohman et al., 2012; Vukovic et al., 2012) than in very old ones, potentially due to reduced glial plasticity. For astrocytes, PE induced hypertrophy and supportive changes (e.g., increased GFAP+ complexity) were robust in middle-aged cohorts (Saur et al., 2014; Fahimi et al., 2017; Belaya et al., 2020), but weaker in aged models (Latimer et al., 2011; Tapia-Rojas et al., 2016). Combined EE+PE showed no clear age specific additive effects (Prado Lima et al., 2018; Singhal et al., 2021). Oligodendrocytes outcomes were limited, but myelin preservation after PE appeared more effective when started earlier (e.g., young adulthood) (Chen et al., 2020; Graciani et al., 2023). Overall, early-to-midlife onset amplified glial benefits, aligning with greater neuroplastic reserve.

• Duration effects

Long-term interventions (≥6 months, started in young adulthood or middle age) amplified effects for both, but EE required longer durations for maximal microglial complexity [e.g., increased ramification after 6–12 months vs. 4–8 weeks (Kumar and Foster, 2007; Ali et al., 2019; Stuart et al., 2019; de Oliveira et al., 2020)]. EE also showed lifespan variations [e.g., stronger in middle-aged vs. aged models (Singhal et al., 2020a,^2021^)], with long-term exposure also enhancing neuroimmune crosstalk (Williamson et al., 2012). Short-term EE (7 weeks) increased microglial number in middle-aged models but not astrocytes (Singhal et al., 2021).

Physical exercise effects were more duration independent for microglia [e.g., reduced activation after 4–6 weeks (Barrientos et al., 2011; Kohman et al., 2012; Vukovic et al., 2012; Lu et al., 2017)], but astrocytic remodeling required longer exposure [e.g., ≥6 months for hypertrophy (Latimer et al., 2011; Belaya et al., 2020)]. Early life onset (young adulthood) enhanced consistency for both, while late life interventions (aged models) yielded smaller magnitudes, potentially due to reduced glial plasticity (He et al., 2017; Stuart et al., 2019; Yan et al., 2023). Sex-specific analyses were limited, but available data suggested stronger PE effects in females [e.g., reversed T-cell accumulation (Chauquet et al., 2024)].

Combined EE+PE yielded variable glial responses regardless of duration, with no evidence of superiority over single modalities (Prado Lima et al., 2018; Singhal et al., 2021). For oligodendrocytes, long-term PE showed consistent anti-demyelinating effects (Chen et al., 2020; Graciani et al., 2023), while short-term data were sparse. Duration dependence suggests cumulative benefits, but heterogeneity in protocols limited direct comparisons.

• Sex differences

Sex specific analyses were underrepresented, constraining generalizability. Where reported, PE effects appeared stronger in females, such as reversed age-related T-cell accumulation and microglial activation in the hippocampus (Chauquet et al., 2024). EE showed no clear sex biases in glial outcomes, though one study noted modest differences in astrocytic responses (Bento-Torres et al., 2017). Combined interventions lacked sex stratified data (Prado Lima et al., 2018; Singhal et al., 2021). Limited evidence suggests potential sex moderation, with females possibly benefiting more from PE due to hormonal influences, but most studies pooled sexes or used single-sex cohorts (predominantly males), highlighting a key gap.

## 4 Discussion

**FIGURE 2:**
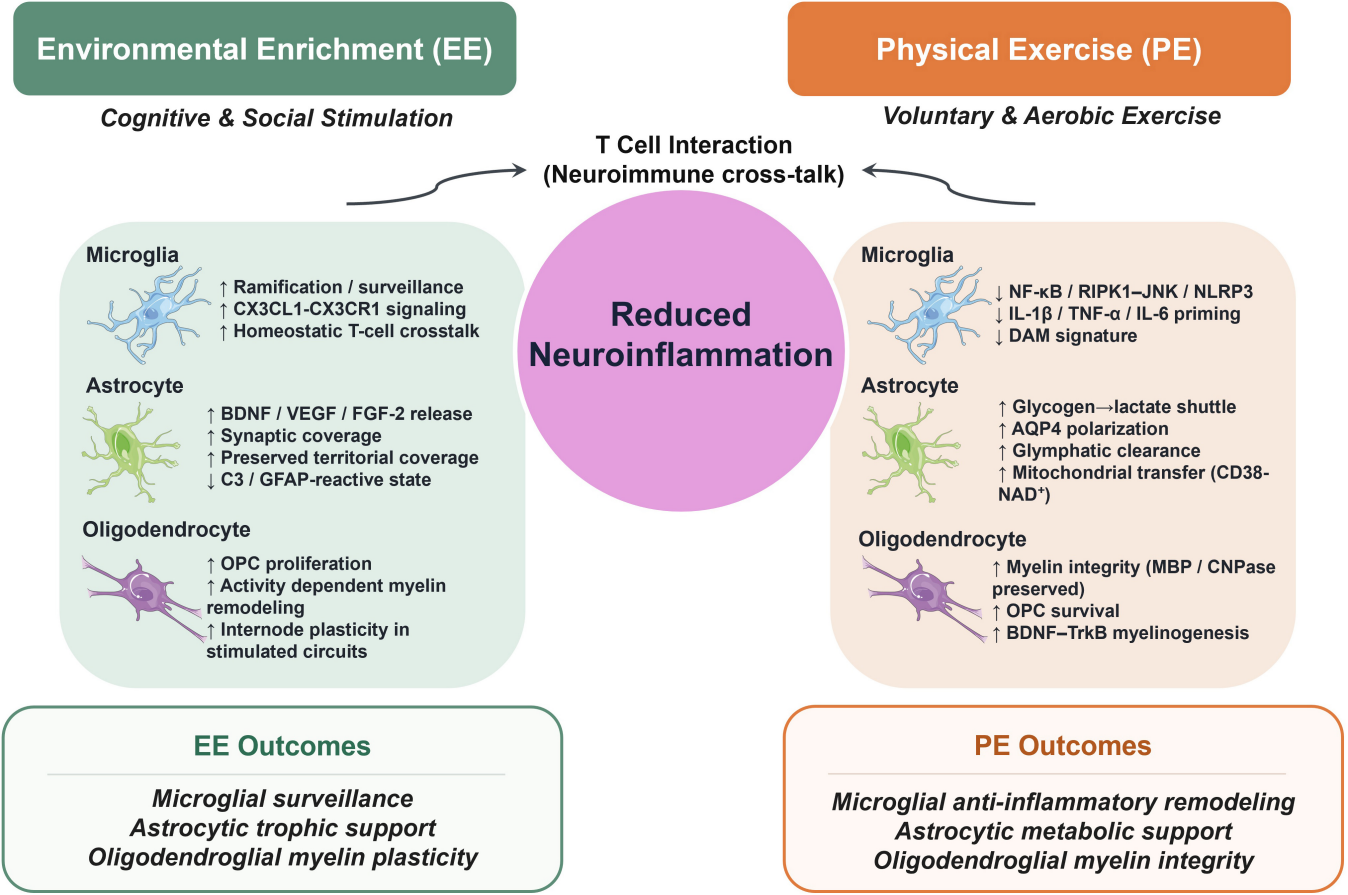
Comparative effects of environmental enrichment and physical exercise on glial cell biology in the aging brain. Schematic comparison of the cell type-specific glial outcomes associated with environmental enrichment (EE) and physical exercise (PE) in aging and aging-relevant rodent models. Microglia: EE preserves surveillance-associated morphology, ramification, CX3CL1-CX3CR1 signaling, and homeostatic T-cell crosstalk. PE more consistently downregulates inflammatory signaling pathways, reduces pro-inflammatory cytokine priming and partially reverses disease-associated microglial signatures. Astrocytes: EE acts predominantly on trophic and synapse-supportive functions, including BDNF, VEGF, and FGF-2 release, preserved synaptic and territorial coverage, and reduced reactive astrocyte signatures. PE acts predominantly on metabolic support functions, including glycogen-lactate shuttling, AQP4 polarization, glymphatic clearance, and mitochondrial transfer. Oligodendrocytes: EE promotes oligodendrocyte precursor cell proliferation, activity-dependent myelin remodeling, and internode plasticity in stimulated circuits. PE supports myelin integrity, OPC survival, and BDNF-TrkB-associated myelinogenesis. The central node (“reduced neuroinflammation”) indicates a convergent endpoint to which both interventions contribute, modulated in part by T cell-glia neuroimmune interactions. CX3CL1-CX3CR1, fractalkine ligand receptor signaling axis; BDNF, brain derived neurotrophic factor; VEGF, vascular endothelial growth factor; FGF-2, fibroblast growth factor-2; C3, complement component 3; GFAP, glial fibrillary acidic protein; NF-kB, nuclear factor kappa B; RIPK1, receptor interacting protein kinase 1; JNK, c-Jun N-terminal kinase; NLRP3, NOD-, LRR- and pyrin domain-containing protein 3; IL, interleukin; TNF-a, tumor necrosis factor alpha; DAM, disease associated microglia; AQP4, aquaporin-4; NAD+, nicotinamide adenine dinucleotide; MBP, myelin basic protein; OPC, oligodendrocyte precursor cell; TrkB, tropomyosin receptor kinase B.

This systematic review synthesized preclinical evidence comparing the effects of EE, PE, and their combination on glial cell dynamics during brain aging. Both interventions exerted beneficial but partially distinct effects on microglia and astrocytes, with limited data available for oligodendrocytes (Figure 2).

Environmental enrichment consistently increased microglial number and morphological complexity (ramification and process length) and promoted a shift toward homeostatic phenotypes, particularly after long-term exposure initiated in middle age (Williamson et al., 2012; Singhal et al., 2014, 2021; Xu et al., 2016; Ali et al., 2019; de Oliveira et al., 2020). EE also modulated neuroimmune crosstalk, including peripheral T-cell subsets in cervical lymph nodes (Williamson et al., 2012; Singhal et al., 2021). In contrast, PE more reliably reversed age-related microglial gene expression changes, attenuated pro-inflammatory signaling (e.g., NF-κB, RIPK1/JNK pathways), and induced adaptive astrocytic hypertrophy and supportive remodeling (increased process complexity and territorial coverage) (Barrientos et al., 2011; Latimer et al., 2011; Vukovic et al., 2012; Saur et al., 2014; Littlefield et al., 2015; Rodriguez et al., 2015; Yoo et al., 2015; Tapia-Rojas et al., 2016; Fahimi et al., 2017; He et al., 2017; Lu et al., 2017; Stevenson et al., 2018; Lundquist et al., 2019; Belaya et al., 2020; Mela et al., 2020; Chen et al., 2021; Cao et al., 2022; Kelty et al., 2022; Liu et al., 2023, 2024; Yamaguchi et al., 2023; Chauquet et al., 2024; Krejcova et al., 2024; Li et al., 2024; Cai et al., 2025). Astrocytic responses to EE were generally weaker and more variable, primarily involving reduced pathological reactivity in disease models (Soffie et al., 1999; Diniz et al., 2010, 2016; Beauquis et al., 2013; Sampedro-Piquero et al., 2014; Bento-Torres et al., 2017).

Combined EE+PE interventions produced additive benefits on neurogenesis and some behavioral outcomes but yielded variable or non-superior effects on glial modulation compared with single modalities (Prado Lima et al., 2018; Singhal et al., 2021). Oligodendrocytes and myelin outcomes were understudied, however PE showed modest but more consistent protective effects against age-related demyelination than EE (Pusic and Kraig, 2014; Jensen et al., 2018; Chen et al., 2020; Graciani et al., 2023; Maugeri et al., 2023). Overall, EE appeared particularly effective at enhancing glial-immune interfaces, whereas PE excelled in metabolic and anti-inflammatory glial remodeling. These differential effects were moderated by age at intervention onset, duration, and brain region, with the hippocampus (especially dentate gyrus) being the most responsive area. Accordingly, the strongest comparative inferences come from physiological aging microglial outcomes, whereas several astrocytic and mechanistic interpretations remain more dependent on disease, injury, or stress-based models.

Glial senescence and age-related neuroinflammation establish the cellular context within which EE and PE act and are therefore central to any comparison of their mechanisms. Senescent glia accumulate lipid droplets, display SA-β-gal activity, undergo epigenetic remodeling, and shift their secretome toward a pro-inflammatory senescence associated secretory phenotype (SASP), comprising IL-6, IL-1β, TNF-α, and chemokines, that propagates senescence to neighboring cells (Sikora et al., 2021; Sukhorukov et al., 2021). In parallel, aged microglia drive astrocyte reactivity through IL-1, TNF and C1q signaling (Clarke et al., 2018), the CX3CL1–CX3CR1 axis becomes dysregulated by elevated neuronal cathepsin S (Liu et al., 2025), TGF-β Smad3 anti-inflammatory feedback is impaired (von Bernhardi et al., 2015), and NLRP3 inflammasome activation becomes exaggerated and prolonged (Tejera et al., 2019). Neuroinflammation is particularly pronounced in white matter, where elevated CD68 and HLA-DR appear even in middle age (Raj et al., 2017). The mechanistic comparison developed below considers EE and PE in this context, recognizing that each intervention must act not only on individual glial populations but on the brain wide inflammatory and senescent milieu that constrains glial responsiveness.

### 4.1 Comparison of mechanisms: shared vs. distinct pathways

Environmental enrichment and PE share several convergent mechanistic nodes, most notably attenuation of chronic neuroinflammation and preservation of neurotrophic support (Ickes et al., 2000; Angelucci et al., 2009; Jurgens and Johnson, 2012; Littlefield et al., 2015; Svensson et al., 2015; Xu et al., 2016; Fahimi et al., 2017; Kelly, 2018; Belaya et al., 2020; Kelty et al., 2022; Liu et al., 2024). Both interventions reduce age-related microglial priming and lower pro-inflammatory cytokines such as IL-1 β and TNF- α, thereby supporting synaptic plasticity and cognitive resilience (Singhal et al., 2014, 2020a,^2021^; Kelly, 2018). However, the initiating drivers appear to differ. EE is best interpreted as an experience-dependent input in which cognitive, sensory, and social complexity alters neuronal activity patterns and neuroimmune communication, with downstream effects on microglial surveillance, territorial remodeling, peripheral T-cell modulation, and microglia-astrocyte crosstalk (Singhal et al., 2014, 2020a,^2021^; Ali et al., 2019; Guo et al., 2022; Nicholson et al., 2022). By contrast, PE is more consistently linked to repeated metabolic and motor activation, which engages CX3CL1-CX3CR1 signaling, mitochondrial remodeling, lactate shuttling, and related anti-inflammatory pathways including suppression of NF-kB and NLRP3-associated signaling (Zhang et al., 2021; Liu et al., 2024; Cai et al., 2025). Viewed in this hierarchy, inflammatory cascades and BDNF-TrkB signaling are more plausibly interpreted as intermediate mediators than universal primary drivers across all paradigms. In several PE studies, restoration of CX3CL1-CX3CR1 signaling or suppression of RIPK1/JNK and NF-kB was associated with lower cytokine burden and reduced senescence-like microglial profiles, whereas in EE studies modulation of peripheral T cells and glial-immune interfaces appears upstream of later structural remodeling (Vukovic et al., 2012; Williamson et al., 2012; Mela et al., 2020; Singhal et al., 2021; Liu et al., 2024). Reduced microglial IL-1beta/TNF-alpha output would, in turn, be expected to lessen secondary astrocytic reactivity, while astrocytic BDNF-related support, metabolic shuttling, and glymphatic regulation likely act further downstream to stabilize synapses and neuronal function (Fahimi et al., 2017; He et al., 2017; Clarke et al., 2018; Cai et al., 2025). On this basis, changes in glial morphology, neurogenesis, and behavior are interpreted here as integrated downstream readouts rather than primary mechanistic events. Because only a subset of studies directly manipulated these pathways, this order remains inferential but provides a clearer framework for interpreting shared versus distinct EE and PE effects.

### 4.2 Implications for glial targeted interventions in brain aging

The findings suggest that EE and PE may differentially influence key aspects of glial biology. EE may be particularly relevant for modulating microglial surveillance and neuroimmune interactions, whereas PE may be more closely associated with anti-inflammatory and metabolic regulation. However, these interpretations are derived from heterogeneous preclinical evidence and should be considered as hypotheses for future translational and clinical investigation rather than direct recommendations.

A more explicit translational framework is also needed to bridge these preclinical findings with human biology. At present, the strongest cross-species signal is not a single pathway but a cluster of glial associated processes that could be followed in human studies through multimodal biomarker panels. These may include neuroimaging proxies of microglial or astrocytic activation, circulating or CSF inflammatory mediators, astrocyte-related proteins such as GFAP, and neurotrophic/metabolic markers linked to plasticity and glial support (Svensson et al., 2015; Kwon and Koh, 2020; Hanslik et al., 2021). In this context, the value of EE and PE may lie less in producing one biomarker specific signature and more in shifting coordinated inflammatory, trophic, and metabolic profiles toward a less age reactive state.

### 4.3 Strengths and limitations of the evidence base

Strengths of this review include its specific focus on glial aging (rather than general neuroplasticity), adherence to PRISMA 2020 guidelines (Page et al., 2021), and comprehensive narrative synthesis of heterogeneous preclinical data. The inclusion of studies with clear glial endpoints (Iba1, GFAP, morphology, cytokine profiles) and the separation of cognitive/sensory versus physical components where possible represent important methodological advances. At the same time, we interpret microglial marker findings in light of the marker used in each study. We observed that Iba1 is widely used marker for microglia, but it is not fully microglia specific, and that more restricted markers such as Tmem119 or P2ry12 were not available in most included datasets. These more restricted markers required contextual interpretation in this review since their expression can vary with aging and disease state (Bennett et al., 2016; Walker et al., 2020).

Key limitations include substantial heterogeneity in intervention protocols, animal strains, ages, durations, and outcome measures, which precluded meta-analysis. Most studies were conducted in male or mixed sex rodents, with limited sex stratified analyses. Only a few investigations directly compared the effects of EE and PE. Many EE paradigms included running wheels, which confounded attribution of effects. Oligodendrocyte and white matter outcomes were markedly underrepresented, and clinical translation remains absent. A structured risk of bias assessment was conducted using the SYRCLE tool, however, the predominance of unclear ratings across several domains reflects limitations in reporting rather than confirmed methodological deficiencies. Another key limitation of the current review is the inclusion of heterogeneous experimental paradigms, including disease and stress models. While these models provide mechanistic insight into glial responses relevant to aging, they limit the extent to which conclusions can be attributed specifically to physiological aging processes. Due to methodological heterogeneity and the narrative synthesis approach, formal statistical subgroup or sensitivity analyses were not performed. Instead, patterns in glial outcomes were explored narratively across key variables, including age at intervention onset, duration, sex, and brain region, based on available data from included studies. These explorations, therefore, highlight inconsistencies, variations, and evidence gaps in how EE, PE, and combined interventions modulate glial dynamics during aging.

### 4.4 Gaps in literature and recommendations for future research

Critical gaps remain in direct comparisons using wheel free EE paradigms, sex stratified analyses, and multiregional assessments that include white matter and oligodendrocytes. Dedicated studies on other understudied glial interfaces, including ependymal cells and the aging neurogenic niche, are also lacking and should be investigated for their responsiveness to EE or PE. Long-term studies starting in early adulthood, combined with advanced techniques (single-cell RNA-seq, senescence specific markers, *in vivo* imaging of glial dynamics), are needed. Future research should also evaluate dose response relationships, optimal intervention timing across the lifespan, and the potential of EE+PE in disease models with prominent glial pathology (e.g., Alzheimer’s, vascular dementia). An additional priority is to determine whether early life EE or PE exposures produce durable neurodevelopmental effects on glial maturation, synaptic pruning, and circuit stabilization that later shape vulnerability or resilience in aging. This may also be relevant to disorders characterized by altered pruning or plasticity, including autism spectrum disorder and seizure susceptibility, where microglial and broader glial regulation of synaptic remodeling is increasingly implicated (Hu et al., 2022; Broer and Pauletti, 2024).

A particularly important gap is the absence of chronic EE and PE studies in aged cortex or white matter that quantify Tmem119 or P2ry12 at protein level. In fact, within the current aged EE and PE literature, the studies have relied primarily on Tmem119 associated or broader single cell transcriptional annotation rather than direct P2ry12 endpoints. For translation to humans, future studies should prioritize biomarker strategies that can be measured across species, including neuroimaging proxies of glial activation, CSF or blood based inflammatory markers, astrocyte associated signals such as GFAP, and neurotrophic or metabolic readouts that reflect glial support functions (Svensson et al., 2015; Kwon and Koh, 2020; Hanslik et al., 2021). Equally important, human studies will need to address biological variability that is only incompletely modeled in rodent work, including sex, frailty, baseline physical activity, comorbidity burden, medication exposure, and life course social and environmental heterogeneity. Translational studies bridging rodent findings to human cohorts will therefore be essential for developing evidence-based, glial targeted lifestyle guidelines to promote healthy brain aging.

## 5 Conclusion

In conclusion, the available literature supports partially distinct effects of EE and PE on glial biology, but the strength of inference differs across model contexts. In physiological aging models, the clearest evidence supports EE related enhancement of microglial surveillance and homeostatic features and PE related attenuation of microglial inflammatory tone, whereas many astrocytic and mechanistic conclusions rely more heavily on disease, injury, or stress paradigms. Combined interventions offer limited additive glial benefits, underscoring the need for more targeted and context specific study designs before broad conclusions can be extended to normative brain aging. Their successful translation, however, will depend on biomarker guided human studies that account for interindividual biological variability rather than assuming uniform responses across aging populations.

## Data Availability

The original contributions presented in this study are included in this article/Supplementary material, further inquiries can be directed to the corresponding author.

## References

[B1] Alzheimers Dementia. (2025). 2025 Alzheimer’s disease facts and figures. *Alzheimer’s Dement.* 21:e70235. 10.1002/alz.70235

[B2] Ali S. Liu X. Queen N. J. Patel R. S. Wilkins R. K. Mo X. et al. (2019). Long-term environmental enrichment affects microglial morphology in middle age mice. *Aging* 11 2388–2402. 10.18632/aging.101923 31039130 PMC6519992

[B3] Angelucci F. De Bartolo P. Gelfo F. Foti F. Cutuli D. Bossu P. et al. (2009). Increased concentrations of nerve growth factor and brain-derived neurotrophic factor in the rat cerebellum after exposure to environmental enrichment. *Cerebellum* 8 499–506. 10.1007/s12311-009-0129-1 19688409

[B4] Ashraf A. Michaelides C. Walker T. A. Ekonomou A. Suessmilch M. Sriskanthanathan A. et al. (2019). Regional distributions of iron, copper and zinc and their relationships with glia in a normal aging mouse model. *Front. Aging Neurosci.* 11:351. 10.3389/fnagi.2019.00351 31920630 PMC6930884

[B5] Barrientos R. M. Frank M. G. Crysdale N. Y. Chapman T. R. Ahrendsen J. T. Day H. E. et al. (2011). Little exercise, big effects: Reversing aging and infection-induced memory deficits, and underlying processes. *J. Neurosci.* 31 11578–11586. 10.1523/JNEUROSCI.2266-11.2011 21832188 PMC3171131

[B6] Beauquis J. Pavia P. Pomilio C. Vinuesa A. Podlutskaya N. Galvan V. et al. (2013). Environmental enrichment prevents astroglial pathological changes in the hippocampus of APP transgenic mice, model of Alzheimer’s disease. *Exp. Neurol.* 239 28–37. 10.1016/j.expneurol.2012.09.009 23022919

[B7] Belaya I. Ivanova M. Sorvari A. Ilicic M. Loppi S. Koivisto H. et al. (2020). Astrocyte remodeling in the beneficial effects of long-term voluntary exercise in Alzheimer’s disease. *J. Neuroinflamm.* 17 271. 10.1186/s12974-020-01935-w 32933545 PMC7493971

[B8] Bennett M. L. Bennett F. C. Liddelow S. A. Ajami B. Zamanian J. L. Fernhoff N. B. et al. (2016). New tools for studying microglia in the mouse and human CNS. *Proc. Natl. Acad. Sci. U. S. A.* 113 E1738–E1746. 10.1073/pnas.1525528113 26884166 PMC4812770

[B9] Bento-Torres J. Sobral L. L. Reis R. R. de Oliveira R. B. Anthony D. C. Vasconcelos P. F. et al. (2017). Age and environment influences on mouse prion disease progression: Behavioral changes and morphometry and stereology of hippocampal astrocytes. *Oxid. Med. Cell Longev.* 2017:4504925. 10.1155/2017/4504925 28243355 PMC5294381

[B10] Bernal G. M. Peterson D. A. (2011). Phenotypic and gene expression modification with normal brain aging in GFAP-positive astrocytes and neural stem cells. *Aging Cell* 10 466–482. 10.1111/j.1474-9726.2011.00694.x 21385309 PMC3094510

[B11] Broer S. Pauletti A. (2024). Microglia and infiltrating macrophages in ictogenesis and epileptogenesis. *Front. Mol. Neurosci.* 17:1404022. 10.3389/fnmol.2024.1404022 38873242 PMC11171130

[B12] Buchhold B. Mogoanta L. Suofu Y. Hamm A. Walker L. Kessler C. et al. (2007). Environmental enrichment improves functional and neuropathological indices following stroke in young and aged rats. *Restor. Neurol. Neurosci.* 25 467–484. 10.3233/RNN-2007-00404 18334765

[B13] Butt T. H. Tobiume M. Re D. B. Kariya S. (2024). Physical exercise counteracts aging-associated white matter demyelination causing cognitive decline. *Aging Dis.* 15 2136–2148. 10.14336/AD.2024.0216 38377028 PMC11346408

[B14] Cai J. Chen Y. She Y. He X. Feng H. Sun H. et al. (2025). Aerobic exercise improves astrocyte mitochondrial quality and transfer to neurons in a mouse model of Alzheimer’s disease. *Brain Pathol.* 35:e13316. 10.1111/bpa.13316 39462160 PMC11961210

[B15] Cao W. Lin J. Xiang W. Liu J. Wang B. Liao W. et al. (2022). Physical exercise-induced astrocytic neuroprotection and cognitive improvement through primary cilia and mitogen-activated protein kinases pathway in rats with chronic cerebral hypoperfusion. *Front. Aging Neurosci.* 14:866336. 10.3389/fnagi.2022.866336 35721009 PMC9198634

[B16] Chauquet S. Willis E. F. Grice L. Harley S. B. R. Powell J. E. Wray N. R. et al. (2024). Exercise rejuvenates microglia and reverses T cell accumulation in the aged female mouse brain. *Aging Cell* 23:e14172. 10.1111/acel.14172 38747044 PMC11258432

[B17] Chen L. Chao F. L. Lu W. Zhang L. Huang C. X. Yang S. et al. (2020). Long-Term running exercise delays age-related changes in white matter in rats. *Front. Aging Neurosci.* 12:590530. 10.3389/fnagi.2020.590530 33192486 PMC7645073

[B18] Chen Z. Gao M. Su Y. Liu P. Sun B. (2021). Running promotes transformation of brain astrocytes into neuroprotective reactive astrocytes and synaptic formation by targeting gpc6 through the STAT3 pathway. *Front. Physiol.* 12:633618. 10.3389/fphys.2021.633618 34122124 PMC8189178

[B19] Cheng G. W. Mok K. K. Yeung S. H. Kofler J. Herrup K. Tse K. H. (2022). Apolipoprotein E epsilon4 mediates myelin breakdown by targeting oligodendrocytes in sporadic Alzheimer disease. *J. Neuropathol. Exp. Neurol.* 81 717–730. 10.1093/jnen/nlac054 35779013 PMC9393713

[B20] Clarke L. E. Liddelow S. A. Chakraborty C. Munch A. E. Heiman M. Barres B. A. (2018). Normal aging induces A1-like astrocyte reactivity. *Proc. Natl. Acad. Sci. U. S. A.* 115 E1896–E1905. 10.1073/pnas.1800165115 29437957 PMC5828643

[B21] Conde J. R. Streit W. J. (2006). Microglia in the aging brain. *J. Neuropathol. Exp. Neurol.* 65 199–203. 10.1097/01.jnen.0000202887.22082.63 16651881

[B22] Cunha Feio, Leal M. D. Amaral Junior F. L. D. Silva Arruda B. F. D. Kurosawa J. A. A. Vieira A. A. et al. (2023). The masticatory activity interference in quantitative estimation of CA1, CA3 and dentate gyrus hippocampal astrocytes of aged murine models and under environmental stimulation. *Int. J. Mol. Sci.* 24:6529. 10.3390/ijms24076529 37047502 PMC10095286

[B23] de Oliveira T. C. G. Carvalho-Paulo D. de Lima C. M. de Oliveira R. B. Santos Filho C. Diniz D. G. et al. (2020). Long-term environmental enrichment reduces microglia morphological diversity of the molecular layer of dentate gyrus. *Eur. J. Neurosci.* 52 4081–4099. 10.1111/ejn.14920 32726468

[B24] Dimovasili C. Fair A. E. Garza I. R. Batterman K. V. Mortazavi F. Moore T. L. et al. (2023). Aging compromises oligodendrocyte precursor cell maturation and efficient remyelination in the monkey brain. *Geroscience* 45 249–264. 10.1007/s11357-022-00621-4 35930094 PMC9886778

[B25] Diniz D. G. de Oliveira M. A. de Lima C. M. Foro C. A. Sosthenes M. C. Bento-Torres J. et al. (2016). Age, environment, object recognition and morphological diversity of GFAP-immunolabeled astrocytes. *Behav. Brain Funct.* 12:28. 10.1186/s12993-016-0111-2 27719674 PMC5056502

[B26] Diniz D. G. Foro C. A. Rego C. M. Gloria D. A. de Oliveira F. R. Paes J. M. et al. (2010). Environmental impoverishment and aging alter object recognition, spatial learning, and dentate gyrus astrocytes. *Eur. J. Neurosci.* 32 509–519. 10.1111/j.1460-9568.2010.07296.x 20704596

[B27] Ehninger D. Kempermann G. (2003). Regional effects of wheel running and environmental enrichment on cell genesis and microglia proliferation in the adult murine neocortex. *Cereb. Cortex* 13 845–851. 10.1093/cercor/13.8.845 12853371

[B28] Fahimi A. Baktir M. A. Moghadam S. Mojabi F. S. Sumanth K. McNerney M. W. et al. (2017). Physical exercise induces structural alterations in the hippocampal astrocytes: Exploring the role of BDNF-TrkB signaling. *Brain Struct. Funct.* 222 1797–1808. 10.1007/s00429-016-1308-8 27686571

[B29] Forbes T. A. Gallo V. (2017). All wrapped up: Environmental effects on myelination. *Trends Neurosci.* 40 572–587. 10.1016/j.tins.2017.06.009 28844283 PMC5671205

[B30] Gao Z. K. Shen X. Y. Han Y. Guo Y. S. Yuan M. Bi X. (2022). Enriched environment effects on myelination of the central nervous system: Role of glial cells. *Neural Plast* 2022:5766993. 10.1155/2022/5766993 35465398 PMC9023233

[B31] Garcia-Dominguez M. (2025). Interplay between aging and glial cell dysfunction: Implications for CNS health. *Life* 15:1498. 10.3390/life15101498 41157171 PMC12565160

[B32] Goldstein E. Z. Pertsovskaya V. Forbes T. A. Dupree J. L. Gallo V. (2021). Prolonged environmental enrichment promotes developmental myelination. *Front. Cell Dev. Biol.* 9:665409. 10.3389/fcell.2021.665409 33981706 PMC8107367

[B33] Graciani A. L. Gutierre M. U. Coppi A. A. Arida R. M. Gutierre R. C. (2023). Myelin, aging, and physical exercise. *Neurobiol. Aging* 127 70–81. 10.1016/j.neurobiolaging.2023.03.009 37116408

[B34] Groh J. Simons M. (2025). White matter aging and its impact on brain function. *Neuron* 113 127–139. 10.1016/j.neuron.2024.10.019 39541972

[B35] Guo Y. S. Yuan M. Han Y. Shen X. Y. Gao Z. K. Bi X. (2022). Effects of enriched environment on microglia and functional white matter recovery in rats with post stroke cognitive impairment. *Neurochem. Int.* 154:105295. 10.1016/j.neuint.2022.105295 35121010

[B36] Hanslik K. L. Marino K. M. Ulland T. K. (2021). Modulation of glial function in health, aging, and neurodegenerative disease. *Front. Cell Neurosci.* 15:718324. 10.3389/fncel.2021.718324 34531726 PMC8439422

[B37] He X. F. Liu D. X. Zhang Q. Liang F. Y. Dai G. Y. Zeng J. S. et al. (2017). Voluntary exercise promotes glymphatic clearance of amyloid beta and reduces the activation of astrocytes and microglia in aged mice. *Front. Mol. Neurosci.* 10:144. 10.3389/fnmol.2017.00144 28579942 PMC5437122

[B38] Hooijmans C. R. Rovers M. M. de Vries R. B. Leenaars M. Ritskes-Hoitinga M. Langendam M. W. (2014). SYRCLE’s risk of bias tool for animal studies. *BMC Med. Res. Methodol.* 14:43. 10.1186/1471-2288-14-43 24667063 PMC4230647

[B39] Hu C. Li H. Li J. Luo X. Hao Y. (2022). Microglia: Synaptic modulator in autism spectrum disorder. *Front. Psychiatry* 13:958661. 10.3389/fpsyt.2022.958661 36465285 PMC9714329

[B40] Ickes B. R. Pham T. M. Sanders L. A. Albeck D. S. Mohammed A. H. Granholm A. C. (2000). Long-term environmental enrichment leads to regional increases in neurotrophin levels in rat brain. *Exp. Neurol.* 164 45–52. 10.1006/exnr.2000.7415 10877914

[B41] Jensen S. K. Michaels N. J. Ilyntskyy S. Keough M. B. Kovalchuk O. Yong V. W. (2018). Multimodal enhancement of remyelination by exercise with a pivotal role for oligodendroglial PGC1alpha. *Cell Rep.* 24 3167–3179. 10.1016/j.celrep.2018.08.060 30232000

[B42] Jurgens H. A. Johnson R. W. (2012). Environmental enrichment attenuates hippocampal neuroinflammation and improves cognitive function during influenza infection. *Brain Behav. Immun.* 26 1006–1016. 10.1016/j.bbi.2012.05.015 22687335 PMC3454448

[B43] Kaya T. Mattugini N. Liu L. Ji H. Cantuti-Castelvetri L. Wu J. et al. (2022). CD8(+) T cells induce interferon-responsive oligodendrocytes and microglia in white matter aging. *Nat. Neurosci.* 25 1446–1457. 10.1038/s41593-022-01183-6 36280798 PMC9630119

[B44] Kelly A. M. (2018). Exercise-Induced modulation of neuroinflammation in models of Alzheimer’s disease. *Brain Plast* 4 81–94. 10.3233/BPL-180074 30564548 PMC6296260

[B45] Kelty T. J. Mao X. Kerr N. R. Childs T. E. Ruegsegger G. N. Booth F. W. (2022). Resistance-exercise training attenuates LPS-induced astrocyte remodeling and neuroinflammatory cytokine expression in female Wistar rats. *J. Appl. Physiol.* 132 317–326. 10.1152/japplphysiol.00571.2021 34941437

[B46] Kohman R. A. Bhattacharya T. K. Kilby C. Bucko P. Rhodes J. S. (2013a). Effects of minocycline on spatial learning, hippocampal neurogenesis and microglia in aged and adult mice. *Behav. Brain Res.* 242 17–24. 10.1016/j.bbr.2012.12.032 23274840 PMC3725815

[B47] Kohman R. A. Bhattacharya T. K. Wojcik E. Rhodes J. S. (2013b). Exercise reduces activation of microglia isolated from hippocampus and brain of aged mice. *J. Neuroinflamm.* 10:114. 10.1186/1742-2094-10-114 24044641 PMC3848770

[B48] Kohman R. A. DeYoung E. K. Bhattacharya T. K. Peterson L. N. Rhodes J. S. (2012). Wheel running attenuates microglia proliferation and increases expression of a proneurogenic phenotype in the hippocampus of aged mice. *Brain Behav. Immun.* 26 803–810. 10.1016/j.bbi.2011.10.006 22056294 PMC3275652

[B49] Krejcova L. V. Bento-Torres J. Diniz D. G. Pereira A. Jr. Batista-de-Oliveira M. de Morais A. et al. (2024). Unraveling the influence of litter size, maternal care, exercise, and aging on neurobehavioral plasticity and dentate gyrus microglia dynamics in male rats. *Brain Sci.* 14:497. 10.3390/brainsci14050497 38790475 PMC11119659

[B50] Kumar A. Foster T. (2007). Environmental enrichment decreases the afterhyperpolarization in senescent rats. *Brain Res.* 1130 103–107. 10.1016/j.brainres.2006.10.037 17169341 PMC1800885

[B51] Kwon H. S. Koh S. H. (2020). Neuroinflammation in neurodegenerative disorders: The roles of microglia and astrocytes. *Transl. Neurodegener.* 9:42. 10.1186/s40035-020-00221-2 33239064 PMC7689983

[B52] Latimer C. S. Searcy J. L. Bridges M. T. Brewer L. D. Popovic J. Blalock E. M. et al. (2011). Reversal of glial and neurovascular markers of unhealthy brain aging by exercise in middle-aged female mice. *PLoS One* 6:e26812. 10.1371/journal.pone.0026812 22046366 PMC3201977

[B53] Li S. Wang H. Qin M. Huang W. Gao H. Song X. et al. (2025). Environmental enrichment attenuates social isolation-exacerbated postoperative cognitive dysfunction in aged mice via inhibition of RAGE-HMGB1 proinflammatory signaling. *Brain Res. Bull.* 229:111462. 10.1016/j.brainresbull.2025.111462 40653061

[B54] Li Y. Luo Y. Zhu P. Liang X. Li J. Dou X. et al. (2024). Running exercise improves astrocyte loss, morphological complexity and astrocyte-contacted synapses in the hippocampus of CUS-induced depression model mice. *Pharmacol. Biochem. Behav.* 239:173750. 10.1016/j.pbb.2024.173750 38494007

[B55] Littlefield A. M. Setti S. E. Priester C. Kohman R. A. (2015). Voluntary exercise attenuates LPS-induced reductions in neurogenesis and increases microglia expression of a proneurogenic phenotype in aged mice. *J. Neuroinflamm.* 12:138. 10.1186/s12974-015-0362-0 26224094 PMC4518639

[B56] Liu P. P. Liu X. H. Ren M. J. Liu X. T. Shi X. Q. Li M. L. et al. (2025). Neuronal cathepsin S increases neuroinflammation and causes cognitive decline via CX3CL1-CX3CR1 axis and JAK2-STAT3 pathway in aging and Alzheimer’s disease. *Aging Cell* 24:e14393. 10.1111/acel.14393 39453382 PMC11822647

[B57] Liu Y. Meng X. Tang C. Zheng L. Tao K. Guo W. (2024). Aerobic exercise modulates RIPK1-mediated MAP3K5/JNK and NF-kappaB pathways to suppress microglia activation and neuroinflammation in the hippocampus of D-gal-induced accelerated aging mice. *Physiol. Behav.* 286:114676. 10.1016/j.physbeh.2024.114676 39181380

[B58] Liu Y. Shen X. Zhang Y. Zheng X. Cepeda C. Wang Y. et al. (2023). Interactions of glial cells with neuronal synapses, from astrocytes to microglia and oligodendrocyte lineage cells. *Glia* 71 1383–1401. 10.1002/glia.24343 36799296

[B59] Lu Y. Dong Y. Tucker D. Wang R. Ahmed M. E. Brann D. et al. (2017). Treadmill exercise exerts neuroprotection and regulates microglial polarization and oxidative stress in a streptozotocin-induced rat model of sporadic Alzheimer’s disease. *J. Alzheimers Dis.* 56 1469–1484. 10.3233/JAD-160869 28157094 PMC5450951

[B60] Lundquist A. J. Parizher J. Petzinger G. M. Jakowec M. W. (2019). Exercise induces region-specific remodeling of astrocyte morphology and reactive astrocyte gene expression patterns in male mice. *J. Neurosci. Res.* 97 1081–1094. 10.1002/jnr.24430 31175682

[B61] Mansour H. Chamberlain C. G. Weible M. W. II Hughes S. Chu Y. Chan-Ling T. (2008). Aging-related changes in astrocytes in the rat retina: Imbalance between cell proliferation and cell death reduces astrocyte availability. *Aging Cell* 7 526–540. 10.1111/j.1474-9726.2008.00402.x 18489730

[B62] Matejuk A. Ransohoff R. M. (2020). Crosstalk between astrocytes and microglia: An overview. *Front. Immunol.* 11:1416. 10.3389/fimmu.2020.01416 32765501 PMC7378357

[B63] Maugeri G. D’Agata V. Musumeci G. (2023). Role of exercise in the brain: Focus on oligodendrocytes and remyelination. *Neural Regen. Res.* 18 2645–2646. 10.4103/1673-5374.373683 37449603 PMC10358674

[B64] Maugeri G. D’Agata V. Magri B. Roggio F. Castorina A. Ravalli S. et al. (2021). Neuroprotective effects of physical activity via the adaptation of astrocytes. *Cells* 10:1542. 10.3390/cells10061542 34207393 PMC8234474

[B65] Mee-Inta O. Zhao Z. W. Kuo Y. M. (2019). Physical exercise inhibits inflammation and microglial activation. *Cells* 8:691. 10.3390/cells8070691 31324021 PMC6678635

[B66] Mela V. Mota B. C. Milner M. McGinley A. Mills K. H. G. Kelly A. M. et al. (2020). Exercise-induced re-programming of age-related metabolic changes in microglia is accompanied by a reduction in senescent cells. *Brain Behav. Immun.* 87 413–428. 10.1016/j.bbi.2020.01.012 31978523

[B67] Miyamoto N. Pham L. D. Hayakawa K. Matsuzaki T. Seo J. H. Magnain C. et al. (2013). Age-related decline in oligodendrogenesis retards white matter repair in mice. *Stroke* 44 2573–2578. 10.1161/STROKEAHA.113.001530 23881957 PMC3818687

[B68] Mrak R. E. Griffin W. S. (2005). Glia and their cytokines in progression of neurodegeneration. *Neurobiol. Aging* 26 349–354. 10.1016/j.neurobiolaging.2004.05.010 15639313

[B69] Nagele R. G. Wegiel J. Venkataraman V. Imaki H. Wang K. C. Wegiel J. (2004). Contribution of glial cells to the development of amyloid plaques in Alzheimer’s disease. *Neurobiol. Aging* 25 663–674. 10.1016/j.neurobiolaging.2004.01.007 15172746

[B70] Nicholson M. Wood R. J. Gonsalvez D. G. Hannan A. J. Fletcher J. L. Xiao J. et al. (2022). Remodelling of myelinated axons and oligodendrocyte differentiation is stimulated by environmental enrichment in the young adult brain. *Eur. J. Neurosci.* 56 6099–6114. 10.1111/ejn.15840 36217300 PMC10092722

[B71] Norden D. M. Godbout J. P. (2013). Review: Microglia of the aged brain: Primed to be activated and resistant to regulation. *Neuropathol. Appl. Neurobiol.* 39 19–34. 10.1111/j.1365-2990.2012.01306.x 23039106 PMC3553257

[B72] Ogrodnik M. Zhu Y. Langhi L. G. Tchkonia T. Krüger P. Fielder E. et al. (2019). Obesity-induced cellular senescence drives anxiety and impairs neurogenesis. *Cell Metab.* 29 1061–1077.e8. 10.1016/j.cmet.2018.12.008. 30612898 PMC6509403

[B73] Page M. J. McKenzie J. E. Bossuyt P. M. Boutron I. Hoffmann T. C. Mulrow C. D. et al. (2021). The PRISMA 2020 statement: An updated guideline for reporting systematic reviews. *BMJ* 372:n71. 10.1136/bmj.n71 33782057 PMC8005924

[B74] Perez R. F. Tezanos P. Penarroya A. Gonzalez-Ramon A. Urdinguio R. G. Gancedo-Verdejo J. et al. (2024). A multiomic atlas of the aging hippocampus reveals molecular changes in response to environmental enrichment. *Nat. Commun.* 15:5829. 10.1038/s41467-024-49608-z 39013876 PMC11252340

[B75] Peters A. (2009). The effects of normal aging on myelinated nerve fibers in monkey central nervous system. *Front. Neuroanat.* 3:11. 10.3389/neuro.05.011.2009 19636385 PMC2713738

[B76] Prado Lima M. G. Schimidt H. L. Garcia A. Dare L. R. Carpes F. P. Izquierdo I. et al. (2018). Environmental enrichment and exercise are better than social enrichment to reduce memory deficits in amyloid beta neurotoxicity. *Proc. Natl. Acad. Sci. U. S. A.* 115 E2403–E2409. 10.1073/pnas.1718435115 29463708 PMC5877978

[B77] Pusic A. D. Kraig R. P. (2014). Youth and environmental enrichment generate serum exosomes containing miR-219 that promote CNS myelination. *Glia* 62 284–299. 10.1002/glia.22606 24339157 PMC4096126

[B78] Raj D. Yin Z. Breur M. Doorduin J. Holtman I. R. Olah M. et al. (2017). Increased white matter inflammation in aging- and Alzheimer’s disease brain. *Front. Mol. Neurosci.* 10:206. 10.3389/fnmol.2017.00206 28713239 PMC5492660

[B79] Robison L. S. Francis N. Popescu D. L. Anderson M. E. Hatfield J. Xu F. et al. (2020). Environmental enrichment: Disentangling the influence of novelty, social, and physical activity on cerebral amyloid angiopathy in a transgenic mouse model. *Int. J. Mol. Sci.* 21:843. 10.3390/ijms21030843 32012921 PMC7038188

[B80] Rodriguez J. J. Noristani H. N. Verkhratsky A. (2015). Microglial response to Alzheimer’s disease is differentially modulated by voluntary wheel running and enriched environments. *Brain Struct. Funct.* 220 941–953. 10.1007/s00429-013-0693-5 24374506

[B81] Rozovsky I. Finch C. E. Morgan T. E. (1998). Age-related activation of microglia and astrocytes: In vitro studies show persistent phenotypes of aging, increased proliferation, and resistance to down-regulation. *Neurobiol. Aging* 19 97–103. 10.1016/s0197-4580(97)00169-3 9562510

[B82] Sampedro-Piquero P. De Bartolo P. Petrosini L. Zancada-Menendez C. Arias J. L. Begega A. (2014). Astrocytic plasticity as a possible mediator of the cognitive improvements after environmental enrichment in aged rats. *Neurobiol. Learn Mem.* 114 16–25. 10.1016/j.nlm.2014.04.002 24727294

[B83] Sams E. C. (2021). Oligodendrocytes in the aging brain. *Neuronal Signal* 5 NS20210008. 10.1042/NS20210008 34290887 PMC8264650

[B84] Saur L. Baptista P. P. de Senna P. N. Paim M. F. do Nascimento P. Ilha J. et al. (2014). Physical exercise increases GFAP expression and induces morphological changes in hippocampal astrocytes. *Brain Struct. Funct.* 219 293–302. 10.1007/s00429-012-0500-8 23288255

[B85] Schlett J. S. Mettang M. Skaf A. Schweizer P. Errerd A. Mulugeta E. A. et al. (2023). NF-kappaB is a critical mediator of post-mitotic senescence in oligodendrocytes and subsequent white matter loss. *Mol. Neurodegener.* 18:24. 10.1186/s13024-023-00616-5 37069623 PMC10108549

[B86] Sikora E. Bielak-Zmijewska A. Dudkowska M. Krzystyniak A. Mosieniak G. Wesierska M. et al. (2021). Cellular senescence in brain aging. *Front. Aging Neurosci.* 13:646924. 10.3389/fnagi.2021.646924 33732142 PMC7959760

[B87] Simpson J. E. Fernando M. S. Clark L. Ince P. G. Matthews F. Forster G. et al. (2007). White matter lesions in an unselected cohort of the elderly: Astrocytic, microglial and oligodendrocyte precursor cell responses. *Neuropathol. Appl. Neurobiol.* 33 410–419. 10.1111/j.1365-2990.2007.00828.x 17442062

[B88] Singhal G. Jaehne E. J. Corrigan F. Baune B. T. (2014). Cellular and molecular mechanisms of immunomodulation in the brain through environmental enrichment. *Front. Cell Neurosci.* 8:97. 10.3389/fncel.2014.00097 24772064 PMC3982075

[B89] Singhal G. Morgan J. Corrigan F. Toben C. Jawahar M. C. Jaehne E. J. et al. (2021). Short-Term environmental enrichment is a stronger modulator of brain glial cells and cervical lymph node T cell subtypes than exercise or combined exercise and enrichment. *Cell Mol. Neurobiol.* 41 469–486. 10.1007/s10571-020-00862-x 32451728 PMC7920895

[B90] Singhal G. Morgan J. Jawahar M. C. Corrigan F. Jaehne E. J. Toben C. et al. (2019a). Short-term environmental enrichment, and not physical exercise, alleviate cognitive decline and anxiety from middle age onwards without affecting hippocampal gene expression. *Cogn. Affect Behav. Neurosci.* 19 1143–1169. 10.3758/s13415-019-00743-x 31463713

[B91] Singhal G. Morgan J. Jawahar M. C. Corrigan F. Jaehne E. J. Toben C. et al. (2020a). Duration of environmental enrichment determines astrocyte number and cervical lymph node T lymphocyte proportions but not the microglial number in middle-aged C57BL/6 mice. *Front. Cell Neurosci.* 14:57. 10.3389/fncel.2020.00057 32256319 PMC7094170

[B92] Singhal G. Morgan J. Jawahar M. C. Corrigan F. Jaehne E. J. Toben C. et al. (2019b). The effects of short-term and long-term environmental enrichment on locomotion, mood-like behavior, cognition and hippocampal gene expression. *Behav. Brain Res.* 368:111917. 10.1016/j.bbr.2019.111917 31004685

[B93] Singhal G. Morgan J. Jawahar M. C. Corrigan F. Jaehne E. J. Toben C. et al. (2020b). Effects of aging on the motor, cognitive and affective behaviors, neuroimmune responses and hippocampal gene expression. *Behav. Brain Res.* 383:112501. 10.1016/j.bbr.2020.112501 31987935

[B94] Sloane J. A. Hinman J. D. Lubonia M. Hollander W. Abraham C. R. (2003). Age-dependent myelin degeneration and proteolysis of oligodendrocyte proteins is associated with the activation of calpain-1 in the rhesus monkey. *J. Neurochem.* 84 157–168. 10.1046/j.1471-4159.2003.01541.x 12485412

[B95] Soffie M. Hahn K. Terao E. Eclancher F. (1999). Behavioural and glial changes in old rats following environmental enrichment. *Behav. Brain Res.* 101 37–49. 10.1016/s0166-4328(98)00139-9 10342398

[B96] Sparkman N. L. Johnson R. W. (2008). Neuroinflammation associated with aging sensitizes the brain to the effects of infection or stress. *Neuroimmunomodulation* 15 323–330. 10.1159/000156474 19047808 PMC2704383

[B97] Stevenson M. E. Lensmire N. A. Swain R. A. (2018). Astrocytes and radial glia-like cells, but not neurons, display a nonapoptotic increase in caspase-3 expression following exercise. *Brain Behav.* 8:e01110. 10.1002/brb3.1110 30240148 PMC6192401

[B98] Strohm A. O. Majewska A. K. (2024). Physical exercise regulates microglia in health and disease. *Front. Neurosci.* 18:1420322. 10.3389/fnins.2024.1420322 38911597 PMC11192042

[B99] Stuart K. E. King A. E. King N. E. Collins J. M. Vickers J. C. Ziebell J. M. (2019). Late-life environmental enrichment preserves short-term memory and may attenuate microglia in male APP/PS1 mice. *Neuroscience* 408 282–292. 10.1016/j.neuroscience.2019.04.015 30999032

[B100] Sukhorukov V. Voronkov D. Baranich T. Mudzhiri N. Magnaeva A. Illarioshkin S. (2021). Impaired mitophagy in neurons and glial cells during aging and age-related disorders. *Int. J. Mol. Sci.* 22:10251. 10.3390/ijms221910251 34638589 PMC8508639

[B101] Svensson M. Lexell J. Deierborg T. (2015). Effects of physical exercise on neuroinflammation, neuroplasticity, neurodegeneration, and behavior: What we can learn from animal models in clinical settings. *Neurorehabil. Neural Repair.* 29 577–589. 10.1177/1545968314562108 25527485

[B102] Tapia-Rojas C. Aranguiz F. Varela-Nallar L. Inestrosa N. C. (2016). Voluntary running attenuates memory loss, decreases neuropathological changes and induces neurogenesis in a mouse model of a Lzheimer’s disease. *Brain Pathol.* 26 62–74. 10.1111/bpa.12255 25763997 PMC8029165

[B103] Tejera D. Mercan D. Sanchez-Caro J. M. Hanan M. Greenberg D. Soreq H. et al. (2019). Systemic inflammation impairs microglial Abeta clearance through NLRP3 inflammasome. *EMBO J.* 38:e101064. 10.15252/embj.2018101064 31359456 PMC6717897

[B104] Tomlinson L. Leiton C. V. Colognato H. (2016). Behavioral experiences as drivers of oligodendrocyte lineage dynamics and myelin plasticity. *Neuropharmacology* 110(Pt B), 548–562. 10.1016/j.neuropharm.2015.09.016 26415537 PMC7863702

[B105] Tremblay M. E. Zettel M. L. Ison J. R. Allen P. D. Majewska A. K. (2012). Effects of aging and sensory loss on glial cells in mouse visual and auditory cortices. *Glia* 60 541–558. 10.1002/glia.22287 22223464 PMC3276747

[B106] Valles S. L. Iradi A. Aldasoro M. Vila J. M. Aldasoro C. de la Torre J. et al. (2019). Function of glia in aging and the brain diseases. *Int. J. Med. Sci.* 16 1473–1479. 10.7150/ijms.37769 31673239 PMC6818212

[B107] Verkhratsky A. (2025). Neuroglial decline defines cognitive ageing. *Ageing Long.* 6 6–21. 10.47855/jal9020-2025-1-2

[B108] von Bernhardi R. Eugenin-von Bernhardi L. Eugenin J. (2015). Microglial cell dysregulation in brain aging and neurodegeneration. *Front. Aging Neurosci.* 7:124. 10.3389/fnagi.2015.00124 26257642 PMC4507468

[B109] Vukovic J. Colditz M. J. Blackmore D. G. Ruitenberg M. J. Bartlett P. F. (2012). Microglia modulate hippocampal neural precursor activity in response to exercise and aging. *J. Neurosci.* 32 6435–6443. 10.1523/JNEUROSCI.5925-11.2012 22573666 PMC6621117

[B110] Walker D. G. Tang T. M. Mendsaikhan A. Tooyama I. Serrano G. E. Sue L. I. et al. (2020). Patterns of expression of purinergic receptor P2RY12, a putative marker for non-activated microglia, in aged and Alzheimer’s disease brains. *Int. J. Mol. Sci.* 21:678. 10.3390/ijms21020678 31968618 PMC7014248

[B111] Williamson L. L. Chao A. Bilbo S. D. (2012). Environmental enrichment alters glial antigen expression and neuroimmune function in the adult rat hippocampus. *Brain Behav. Immun.* 26 500–510. 10.1016/j.bbi.2012.01.003 22281279 PMC3294275

[B112] Windener F. Grewing L. Thomas C. Dorion M. F. Otteken M. Kular L. et al. (2024). Physiological aging and inflammation-induced cellular senescence may contribute to oligodendroglial dysfunction in MS. *Acta Neuropathol.* 147:82. 10.1007/s00401-024-02733-x 38722375 PMC11082024

[B113] Xu H. Gelyana E. Rajsombath M. Yang T. Li S. Selkoe D. (2016). Environmental enrichment potently prevents microglia-mediated neuroinflammation by human amyloid beta-protein oligomers. *J. Neurosci.* 36 9041–9056. 10.1523/JNEUROSCI.1023-16.2016 27581448 PMC5005718

[B114] Yamaguchi N. Sawano T. Nakatani J. Nakano-Doi A. Nakagomi T. Matsuyama T. et al. (2023). Voluntary running exercise modifies astrocytic population and features in the peri-infarct cortex. *IBRO Neurosci. Rep.* 14 253–263. 10.1016/j.ibneur.2023.02.004 36880055 PMC9984846

[B115] Yan J. Liu Y. Zheng F. Lv D. Jin D. (2023). Environmental enrichment enhanced neurogenesis and behavioral recovery after stroke in aged rats. *Aging* 15 9453–9463. 10.18632/aging.205010 37688770 PMC10564416

[B116] Yanguas-Casas N. Crespo-Castrillo A. Arevalo M. A. Garcia-Segura L. M. (2020). Aging and sex: Impact on microglia phagocytosis. *Aging Cell* 19:e13182. 10.1111/acel.13182 32725944 PMC7431836

[B117] Yoo D. Y. Chae J. Jung H. Y. Yim H. S. Kim J. W. Nam S. M. et al. (2015). Treadmill exercise is associated with reduction of reactive microgliosis and pro-inflammatory cytokine levels in the hippocampus of type 2 diabetic rats. *Neurol. Res.* 37 732–738. 10.1179/1743132815Y.0000000015 25797150

[B118] Zarate S. C. Traetta M. E. Codagnone M. G. Seilicovich A. Reines A. G. (2019). Humanin, a mitochondrial-derived peptide released by astrocytes, prevents synapse loss in hippocampal neurons. *Front. Aging Neurosci.* 11:123. 10.3389/fnagi.2019.00123 31214013 PMC6555273

[B119] Zarif H. Nicolas S. Petit-Paitel A. Chabry J. Guyon A. (2018). “How does enriched environment impact hippocampus brain plasticity?,” in *The Hippocampus–Plasticity and Functions*, ed. A. Stuchlik (London: IntechOpen).

[B120] Zhang X. Huang N. Xiao L. Wang F. Li T. (2021). Replenishing the aged brains: Targeting oligodendrocytes and myelination? *Front. Aging Neurosci.* 13:760200. 10.3389/fnagi.2021.760200 34899272 PMC8656359

