# Supplementary material for: Differential effects of environmental enrichment and physical exercise on glial biology in aging and aging-related conditions: a systematic review
Source: Front Cell Neurosci. 2026 Jul 28;20:1859424. doi: 10.3389/fncel.2026.1859424 (PMC13457000; doi:10.3389/fncel.2026.1859424)
Supplement: Supplementary file 1 [file Supplementary_file_1.docx]

**Differential effects of environmental enrichment and physical exercise on glial biology in aging and aging-related conditions: A systematic review**

Gaurav Singhal^1^, Bernhard T Baune^2*^

^1^Department of Otolaryngology, University of Wisconsin - Madison, United States

^2^Department of Mental Health, University of Münster, Münster, Germany

**Running title:** Physical Exercise vs Environmental Enrichment

***Corresponding author**

Prof. Bernhard T. Baune

Professor of Psychiatry, University of Münster, Germany

Chair and Director, University Hospital of Psychiatry

Director, Lab division of Molecular Neurobiology of Mental Health

University of Münster

Münster, Germany

**Email addresses of the authors:**

[gsinghal4@wisc.edu](mailto:gsinghal4@wisc.edu)

[bernhard.baune@uni-muenster.de](mailto:bernhard.baune@uni-muenster.de)

Supplementary Tables: 3

**Supplementary Table 1: Completed PRISMA 2020 checklist for reporting systematic reviews**

| **Section** | **Item #** | **Checklist item** | **Status** | **Location in manuscript** | **Explanation/Justification** |
| --- | --- | --- | --- | --- | --- |
| Title | 1 | Title: Identify the report as a systematic review, meta-analysis, or both. | Yes | Title page | Title explicitly states, "A Systematic Review" |
| Abstract | 2 | Structured summary: Provide a structured summary including background, objectives, data sources, study eligibility criteria, participants and interventions, study appraisal and synthesis methods, results, limitations, conclusions, and implications of key findings. | Yes | Abstract | Abstract includes all required elements (background, objectives, methods, results, conclusions). |
| Introduction | 3 | Rationale: Describe the rationale for the review in the context of what is already known. | Yes | Introduction | Rationale provided on glial aging, EE/PE mechanisms, evidence gaps, and need for comparison. |
| Introduction | 4 | Objectives: Provide an explicit statement of questions being addressed with reference to participants, interventions, comparisons, outcomes, and study design (PICOS). | Yes | Introduction | Objectives stated using PICOS framework (population: aging models; interventions: EE/PE/combined; comparators: controls; outcomes: glial markers; study design: experimental). |
| Methods | 5 | Protocol and registration: Indicate if a review protocol exists, if and where it can be accessed (e.g., Web address), and, if available, provide registration information including registration number. | Yes | Methods | No formal protocol was registered, but methods follow PRISMA 2020. |
| Methods | 6 | Eligibility criteria: Specify study characteristics (e.g., PICOS, length of follow-up) and report characteristics (e.g., years considered, language, publication status) used as criteria for eligibility. | Yes | Methods: Inclusion/Exclusion Criteria | Focus on rodent models, English peer-reviewed articles, glial outcomes prioritized. |
| Methods | 7 | Information sources: Describe all information sources (e.g., databases, registers, grey literature) in the search within each database, including date of the most recent search. | Yes | Methods: Search and selection process | Scopus, PubMed, Google Scholar; last search November 2025; reference lists and citation chasing. |
| Methods | 8 | Search: Present full electronic search strategy for at least one database, including any limits used, such that it could be repeated. | Yes | Methods (supplement) | Keywords listed, full search strings to be added in Supplementary Table. |
| Methods | 9 | Study selection: State the process for selecting studies (e.g., deduplication, screening titles/abstracts, full-text review). | Yes | Methods: Search and selection process | Titles/abstracts screened, full text assessed, duplicates removed in EndNote. |
| Methods | 10 | Data collection process: Specify the methods of data extraction from reports (e.g., piloted forms, independently, in duplicate) and any processes for obtaining and confirming data from investigators. | Yes | Methods | Data extracted independently by one author (GS), data reviewed by second author (BB). |
| Methods | 11 | Data items: List and define all outcomes for which data were sought, specifying whether all results that were compatible with each outcome domain in each study were sought (e.g., all measures, time points, analyses). | Yes | Methods: Inclusion/Exclusion Criteria | Primary: glial markers (e.g., Iba1, GFAP, morphology), Secondary: neuroplasticity/behavior linked to glia. |
| Methods | 12 | Risk of bias in individual studies: Describe methods used for assessing risk of bias of individual studies (including specification of whether this was done at the outcome or study level), and how this information is to be used in any data synthesis. | Yes | Methods | A structured risk-of-bias assessment using the SYRCLE tool was applied across all included experimental studies evaluating the effects of EE and PE on glial outcomes during aging and aging-related conditions. |
| Methods | 13a | Summary measures: State the principal summary measures (e.g., risk ratio, difference in means). | NA | NA | Narrative synthesis only. No quantitative summary measures. |
| Methods | 13b | Synthesis of results: Describe the methods of handling data and combining results of studies, if done, including measures of consistency (e.g., I²) for each meta-analysis. | NA | NA | No meta-analysis conducted due to heterogeneity. |
| Methods | 13c | Risk of bias across studies: Specify any assessment of risk of bias that may affect the cumulative evidence (e.g., publication bias, selective reporting within studies). | Yes | Methods | Risk of bias across studies was assessed through evaluation of selective reporting within individual studies using the SYRCLE risk of bias tool. Formal assessment of publication bias (e.g., funnel plots) was not performed due to the narrative synthesis approach and heterogeneity of included studies. |
| Methods | 13d | Additional analyses: Describe methods of additional analyses (e.g., sensitivity or subgroup analyses, meta-regression), if done, indicating which were pre-specified. | NA | NA | No meta-analysis due to heterogeneity. |
| Results | 14 | Study selection: Give numbers of studies screened, assessed for eligibility, and included in the review, with reasons for exclusions at each stage, ideally with a flow diagram. | Yes | Results (PRISMA flow diagram) | PRISMA flow diagram (Figure 1) to be included. |
| Results | 15 | Study characteristics: For each study, present characteristics for which data were extracted (e.g., study size, PICOS, follow-up period) and provide the citations. | Yes | Results: Study characteristics | Table summary included study characteristics (species, age, intervention details, glial outcomes). |
| Results | 16a | Risk of bias within studies: Present assessments of risk of bias for each included study. | No | NA | Risk of bias is summarized at the domain and outcome level to better support interpretation of the overall evidence. |
| Results | 16b | Risk of bias across studies: Present assessments of risk of bias across studies. | Yes | Results (4.1 SYRCLE risk of bias assessment) | Risk of bias across studies was summarized at the domain level. |
| Results | 17 | Results of individual studies: For all outcomes considered (benefits or harms), present, for each study: (a) simple summary data for each intervention group (b) effect estimates and confidence intervals, ideally with a forest plot. | NA | NA | Narrative synthesis only. No forest plots. |
| Results | 18 | Synthesis of results: Present results of each meta-analysis done, including confidence intervals and measures of consistency. | NA | NA | No meta-analysis. |
| Results | 19 | Risk of bias across studies: Present results of any assessment of risk of bias across studies. | Yes | Results (4.1 SYRCLE risk of bias assessment) | Risk of bias across studies was summarized at the domain level, with most SYRCLE domains rated as unclear due to limited reporting, while baseline characteristics were consistently rated as low risk. |
| Results | 20 | Additional analysis: Give results of additional analyses, if done (e.g., sensitivity or subgroup analyses, meta-regression). | NA | NA | Narrative synthesis only. |
| Discussion | 21 | Summary of evidence: Summarize the main findings including the strength of evidence for each main outcome; consider their relevance to key groups (e.g., health care providers, users, and policy makers). | Yes | Discussion | Summary provided, including strength of evidence for glial outcomes (e.g., moderate for EE on microglia, limited for oligodendrocytes) and relevance to stakeholders like clinicians (lifestyle interventions) and researchers (need for head-to-head direct trials). |
| Discussion | 22 | Limitations: Discuss limitations at study and outcome level (e.g., risk of bias), and at review-level (e.g., incomplete retrieval of identified research, reporting bias). | Yes | Discussion: Limitations | Limitations were discussed at study and outcome levels (heterogeneity, confounding in EE paradigms, and risk of bias due to under reporting) and at the review level (narrative synthesis, preclinical focus, and limited sex specific data). |
| Discussion | 23 | Conclusions: Provide a general interpretation of the results in the context of other evidence, and implications for future research. | Yes | Discussion/ Conclusion | General interpretation (EE and PE have distinct glial benefits) and implications (tailored interventions, research gaps) provided. |
| Funding | 24 | Funding: Describe sources of funding for the systematic review and other support (e.g., supply of data); role of funders for the systematic review. | Yes | Disclosure | No funding, no conflicts |

**Supplementary Table 2: Electronic search strings used in the systematic review**

| **Database** | **Search strings** | **Filters applied** |
| --- | --- | --- |
| **PubMed** | ("environmental enrichment" OR "enriched environment" OR "enriched housing" OR “EE”) AND ("physical exercise" OR "physical activity" OR “exercise” OR "forced exercise" OR "voluntary exercise" OR “running” OR “treadmill” OR “wheel-running” OR "wheel running") AND (“glia*” OR “glial” OR “microglia*” OR “microglial” OR “astrocyt*” OR “astroglial” OR “astrocyte* OR “oligodendrocyte*” OR “oligodendroglial” OR “OPC” OR "oligodendrocyte precursor" OR "NG2 cell") AND (“aging” OR “ageing” OR "brain aging" OR "brain ageing" OR “age-related” OR "aged brain" OR “senescence*” OR "old age" OR "middle-aged" OR "aged animal*" OR "aged rodent*" OR "aged mice" OR "aged rats") | None during initial search. No species filter (to capture both preclinical and clinical data). Article types: No limits (original research prioritized). |
| **Scopus** | Used similar strings as Pubmed but with Scopus field codes (e.g., TITLE-ABS-KEY for title/abstract/keywords). | Same as PubMed, followed by manual review |
| **Google Scholar** | Used similar strings as Pubmed with free text. Example: "environmental enrichment" OR "enriched environment" "physical exercise" OR exercise glia OR microglia OR astrocyte aging OR "brain aging" | Same as PubMed, followed by manual review |

**Supplementary Table 3: SYRCLE domain descriptions and rating scale**

| **Domain** | **Domain Name** | **Bias Category** | **Assessment Question** |
| --- | --- | --- | --- |
| D1 | Sequence generation | Selection | Was the allocation sequence adequately generated? |
| D2 | Baseline characteristics | Selection | Were the groups similar at baseline (age, sex, weight, health status)? |
| D3 | Allocation concealment | Selection | Was the allocation sequence adequately concealed until animals were enrolled? |
| D4 | Random housing | Performance | Were animals randomly housed during the experiment? |
| D5 | Blinding of caregivers/investigators | Performance | Were caregivers and/or investigators blinded to the intervention? |
| D6 | Random outcome assessment | Detection | Were animals randomly selected for outcome assessment? |
| D7 | Blinding of outcome assessors | Detection | Were outcome assessors blinded to group allocation when measuring outcomes? |
| D8 | Incomplete outcome data | Attrition | Were incomplete outcome data adequately addressed? |
| D9 | Selective outcome reporting | Reporting | Was the study apparently free of selective outcome reporting? |
| D10 | Other sources of bias | Other | Was the study apparently free of other potential sources of bias? |
